# Graphitic carbon nitride nanotubes: a new material for emerging applications†

**DOI:** 10.1039/d0ra05580h

**Published:** 2020-09-15

**Authors:** Oleksandr Stroyuk, Oleksandra Raievska, Dietrich R. T. Zahn

**Affiliations:** a Forschungszentrum Jülich GmbH, Helmholtz-Institut Erlangen Nürnberg für Erneuerbare Energien (HI ERN) Immerwahrstr. 2 91058 Erlangen Germany o.stroyuk@fz-juelich.de; b L.V. Pysarzhevsky Institute of Physical Chemistry, Nat. Acad. of Science of Ukraine 03028 Kyiv Ukraine; c Semiconductor Physics, Chemnitz University of Technology D-09107 Chemnitz Germany zahn@physik.tu-chemnitz.de; d Center for Materials, Architectures, and Integration of Nanomembranes (MAIN), Chemnitz University of Technology D-09107 Chemnitz Germany

## Abstract

We provide a critical review of the current state of the synthesis and applications of nano- and micro-tubes of layered graphitic carbon nitride. This emerging material has a huge potential for light-harvesting applications, including light sensing, artificial photosynthesis, selective photocatalysis, hydrogen storage, light-induced motion, membrane technologies, and can become a major competitor for such established materials as carbon and titania dioxide nanotubes. Graphitic carbon nitride tubes (GCNTs) combine visible-light sensitivity, high charge carrier mobility, and exceptional chemical/photochemical stability, imparting this material with unrivaled photocatalytic activities in photosynthetic processes, such as water splitting and carbon dioxide reduction. The unique geometric GCNT structure and versatility of possible chemical modifications allow new photocatalytic applications of GCNTs to be envisaged including selective photocatalysts of multi-electron processes as well as light-induced and light-directed motion of GCNT-based microswimmers. Closely-packed arrays of aligned GCNTs show great promise as multifunctional membrane materials for the light energy conversion and storage, light-driven pumping of liquids, selective adsorption, and electrochemical applications. These emerging applications require synthetic routes to GCNTs with highly controlled morphological parameters and composition to be available. We recognize three major strategies for the GCNT synthesis including templating, supramolecular assembling of precursors, and scrolling of nano-/microsheets, and outline promising routes for further progress of these approaches in the light of the most important emerging applications of GCNTs.

## Abstract 2

The current state of the synthesis and applications of nano- and micro-tubes of graphitic carbon nitride is critically reviewed.

## Graphical abstract

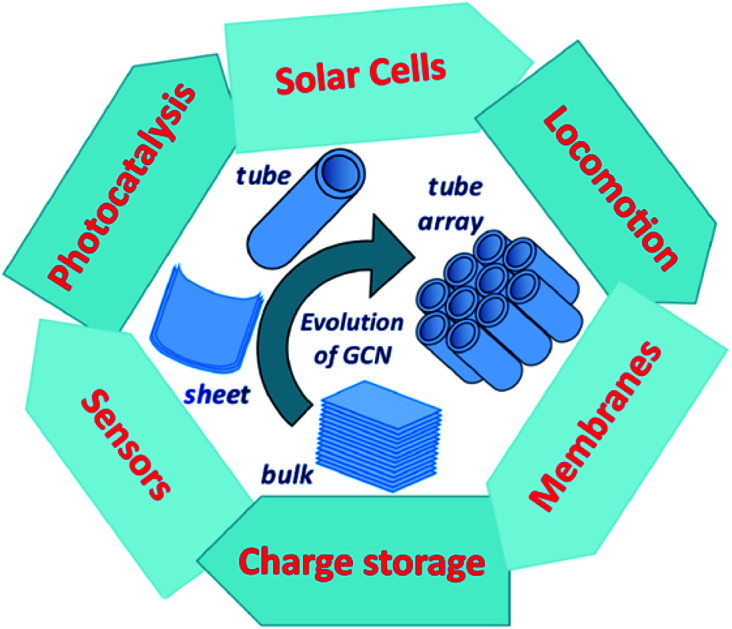

## 1 Introduction

A quest for new functional materials and new properties of conventional materials stimulated broad inter-disciplinary scientific fertilization and a lot of new knowledge has recently been generated combining two and more disciplines, such as physics, biology, materials science, and chemistry. In the two latter disciplines crossing the boundaries has resulted in new materials combining organic and inorganic building blocks as well as entirely organic substances manifesting semiconductor behavior typically associated with inorganic materials (below we provide references to some of the most recent reviews for specific topics). The first trend can be vividly exemplified by hybrid organic–inorganic perovskites showing an explosive growth of recognition in the last decade^1–7^ as well as by metal–organic frameworks, which combine almost unlimited variability of composition and structure with distinct functional properties and perspectives in optics, sensorics, and catalysis.^8–11^ The second trend has brought to the broad attention a layered organic polymeric semiconductor – graphitic carbon nitride (GCN)^12–21^ and stimulated a constant search for new types of organic frameworks with highly tailored functional properties.^22–26^

GCN has a unique fate, being probably the oldest artificially synthesized organic polymer in the middle of 19^th^ century and remaining unrecognized almost till the end of 20^th^ century, when it gradually gained attention as a promising heterogeneous catalyst of hydrogenation/dehydrogenation reactions.^12,13,15,27,28^ Similar to the hybrid perovskites, which were first reported as spectral sensitizers in liquid-junction solar cells, but burst into celebrity in solid-state photovoltaics, GCN rocketed to popularity when it was finally recognized as a visible-light-sensitive photocatalyst for a variety of reactions, including water splitting, CO_2_ conversion, and oxidation of organic compounds.^12–14,16–21,27,29–40^

The positions of the conduction (CB) and valence (VB) band levels of GCN appear to be very favorable for most photocatalytic processes, which, along with the simplicity of the synthesis, low toxicity, and unrivaled photochemical stability, ensured for GCN one of the primary places in the pantheon of semiconductor photocatalysts, along with TiO_2_, ZnO, and CdS. Indeed, GCN shows electronic and light absorption properties very similar to those of cadmium sulfide, but is uncomparably more resistant to photochemical corrosion making it one of the most universal and strongly studied semiconductor photocatalyst of the last decade.^12,13,16,17,19,20,30–35,39,40^ Along with photocatalysis, GCN-based materials and composites found numerous applications in catalysis,^13,15,28,29,38^ electrochemical systems,^13,15,38,41–44^ sensors,^15,43,45^ luminescent materials,^15,43,46^ biomedicine^15,39,43,47,48^ as well as in non-photocatalytic light-harvesting systems.^15,30,43,49^

As for the most photoactive inorganic semiconductors, the photocatalytic activity of GCN is strongly limited by its low specific surface area (3–5 m^2^ g^−1^) and the high rate of electron–hole recombination.^12,15,16,29,43^ In the case of “conventional” inorganic semiconductors, these limitations could be defeated by assembling nanocrystalline semiconductors into mesoporous materials with high surface area as well as by designing single crystals or nanocrystal assemblies with anisotropic shape, including nanorods, nanowires, nanosheets, nanotubes, and hollow spheres, where a directed charge carrier migration can be achieved and electron–hole recombination successfully avoided.^50,51^ These approaches were found to be applicable and very fruitful also for GCN.^12,15–17,21,27,29–35,38,43,47,48,52^ An additional dimension in the design of the GCN-based photocatalytic systems was introduced by the inherent layered structure of GCN, which is formed by ππ-stacked atomically-thick aromatic layers of infinite networks of heptazine (tri-*s*-triazine) or triazine heterocycles. The studies of possible routes of GCN exfoliation^16,21,53,54^ into single and a-few-layer carbon nitride sheets were additionally stimulated by the recent boom in the physics and chemistry of graphene, graphene oxide, and related 2D materials.^55–60^

In contrast to conventional inorganic semiconductors, where new geometries were mostly constructed from nanocrystals (NCs), in the case of GCN both NCs as well as subnanometer and nanometer-thick polyheptazine sheets became available for the development of new shapes and morphologies opening totally new strategies for the material design. A rapid development of the chemistry of exfoliated GCN, thus, stimulated the progress with other geometries including assemblies of 2D GCN sheets into nano- and micrometer rods, wires, spheres, and tubes.^15,16,27,29,31,43^

Semiconductor nanotubes were always credited with a high interest due to the high surface-to-volume ratio, anisotropy of electron transport and optical activity, and special mechanical properties.^43,61–65^ They also serve as a convenient platform for first-principles calculations of electronic effects in 2D materials put under a mechanical stress due to scrolling into tubes of different geometry.^62,66^ In the case of GCN, however, the studies of nano-/micro-tubes have progressed much more slowly than the corresponding studies of nano-/micro-sheets,^15,16,27,29,31,43^ and many unique properties of GCN tubes (GCNTs) remain under-evaluated, still to be discovered and put into applications.

In this view, the aim of our review is to collect available data on GCNTs in a single account and analyze the current state in the design of tubular GCN materials, understanding of their properties and the progress in applications. Our analysis evidences the huge potential of GCNTs for light-harvesting applications, including light sensing, photocatalysis, and photovoltaics, as well as in membrane technologies, adsorption and catalysis, where the GCNTs can become a major competitor for such established materials as carbon and titanium dioxide nanotubes. The graphitic carbon nitride tubes (GCNTs) combine visible-light sensitivity, a high charge carrier mobility, and an exceptional chemical/photochemical stability with large surface area. As a result, GCNTs typically demonstrate high photocatalytic activities in photosynthetic processes, such as water splitting and carbon dioxide reduction, unrivaled by bulk and nanosheet GCNs. The unique geometric GCNT structures and versatility of possible chemical modifications allow new photocatalytic applications of GCNTs to be envisaged, including selective photocatalysts of multi-electron processes as well as light-induced and light-directed motion of GCNT-based microswimmers at the expense of the photocatalytic decomposition of appropriate fuels. Closely-packed arrays of aligned GCNTs show great promise as multifunctional membrane materials for the light energy conversion and storage, the new and emerging applications including photodetection, photocapacitors, selective photocatalysis, and light-driven pumping of liquids, as well as new adsorption and electrochemical processes.

In the first section of this review we discuss the most fruitful and promising strategies for the formation of GCNTs and modification of their morphological, electronic, and optical properties, including template synthesis, synthesis from supramolecular precursor assemblies, as well as scrolling of GCN nanosheets. The second part is dedicated to the established and emerging applications of individual GCNTs and membrane materials formed by close-packed GCNT arrays. In this section we highlight the potential applications of GCNTs predicted by computational methods (such as hydrogen storage), photocatalytic systems based on GCNTs including water splitting, CO_2_ and N_2_ reduction and degradation of organic compounds, and emerging applications of GCNTs as light-steered microswimmers and GCNT membranes – as light-sensitive components of photocapacitors and photodiodes. The review is concluded by an outlook, where we put forth the vision of possible future development in the synthesis of tubular GCN materials as well as their most striking features and feasible advances in their applications.

We note that this review focuses mostly on polyheptazine-based GCNTs with a short description of current research status on the rather rare examples of polytriazine-based GCNTs. Thus, we leave outside the scope the nitrogen-doped carbon nanotubes typically produced by plasma- or synchrotron-assisted chemical vapor deposition with iron catalysts.^67–75^ Such NTs contain 2–3 at% of nitrogen and resemble pristine carbon NTs, for example by the growth mechanism and bamboo-like shape, show no semiconductor behavior or distinct light harvesting properties.

## 2 Synthesis of graphitic carbon nitride tubes

Most of the reports on the formation of GCNTs can be categorized^13,17,78^ into three major strategies: (i) formation of GCNTs by *using templates*, both “hard” ones, such as inorganic membranes with arrays of nanoholes, and “soft” templates, such as polymer globules or ionic liquids; (ii) formation of GCNTs from microrod-shaped precursors formed by *supramolecular assemblies* of two or more types of nitrogen-rich molecules associated by hydrogen bonds; (iii) *scrolling of GCN nano-/microsheets* into tubular formations either in a spontaneous manner or forced by external factors such as thermal treatment, ultrasound, or chemical agents.

### 2.1 Template synthesis of GCNTs

#### “Hard” templates

Synthesis of GCNTs in highly organized templates, such as arrays of nano-/micro-dimensional pores produced by anodic dissolution of aluminum, referred to as anodized aluminum oxide (AAO) membranes^76–80^ proved to be one of the most fruitful approaches to GCN membranes formed by closely packed tubes.

The GCN can be formed directly in the AAO pores by the thermal polycondensation of an appropriate nitrogen-rich precursor (urea, thiourea, dicyandiamide (DCDA) or melamine (M)). Alternatively, the GCN can be deposited onto the walls of AAO in a highly controlled manner by using the inherent properties of melamine to sublime during calcination and undergo polymerization in the gas phase.

The size and shape of AAO membranes can be precisely controlled by adjusting the conditions of electrochemical etching (voltage, current density, sweep rate, electrolyte composition, pore pre-patterning, *etc.*).^77–81^ After the deposition the template can be dissolved by concentrated bases (NaOH, KOH) leaving a GCN replica of the AAO membrane. A cyclic electrochemical etching allows to produce concave pores in AAO^78^ thus opening the possibilities of designing arrays of GCN tubes with a gradual shape. The same effect can be achieved by adjusting the flow conditions during the gas-phase polycondensation and deposition of GCN into the pores of AAO membranes.^82^

Close-packed GCNT membranes were formed using AAO membranes as a template by electron cyclotron resonance chemical vapor deposition (CVD).^83,84^ In this method, a plasma of ionized N_2_ and C_2_H_2_ molecules produced by microwave heating was directed into the pore channels of an AAO membrane, to which a negative bias was applied to promote the plasma flux and the formation of uniform GCNTs. After dissolution of the AAO template the GCN membrane was obtained with a channel diameter of around 250 nm and a thickness of 50–80 μm.^83,84^ With no applied bias, the GCN deposition occurs only on the external side of membrane blocking the entrance to the channels and no tubes can be formed.^84^ The thickness of the GCN layer deposited on the inner AAO walls can be increased by decreasing the flow rate of the gas mixture due to a longer residence time of free radicals within the hollow channels of the AAO membrane.^84^

In ref. 85 the GCNT membranes were produced using AAO membranes as a template by a chemical vapor deposition (CVD) approach combining the sublimation and condensation of melamine into GCN in an argon flow at 520 °C. The channel size of the final GCNT membrane depends on the channel size in the initial AAO and the CVD conditions and varies from around 10 to 75–80 nm.^85^ The AAO template can then be removed by the dissolution in H_3_PO_4_.

The polycondensation of cyanamide at 600 °C inside the channels of an AAO membrane followed by the dissolution of AAO in concentrated NaOH results in the arrays of hollow GCNTs.^86^ By combining the cyanamide condensation with the thermal decomposition of zinc(ii) oxalate the GCN tubes were filled with nanocrystalline ZnO.

**Fig. 1:**
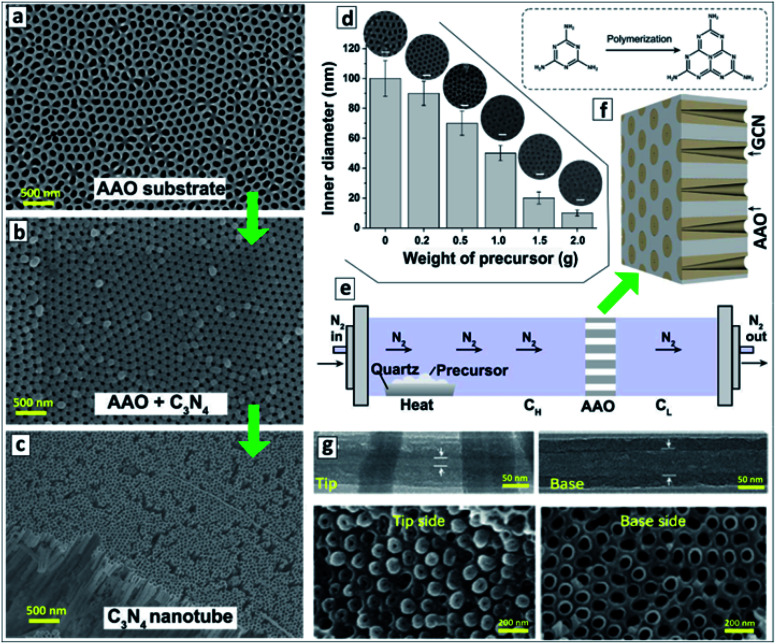
(a–c) SEM images of original AAO membrane (a), GCN deposited onto the AAO membrane (b), and GCNT membrane after the dissolution of AAO (c). Reprinted with permission from ref. 88. Copyright (2020) Elsevier. (d) Relationship between the inner channel diameter of GCN membrane on the weight of precursor used for the synthesis with corresponding SEM images. Reprinted from ref. 87. (e) Scheme of the vapor-deposition reactor designed for the fabrication of asymmetric GCNT membranes. (f) Layout of the asymmetric GCN walls in AAO membrane and a scheme of transformation of melamine into melem as the first polycondensation step leading to the GCN formation. (g) TEM images of a single GCN tube (upper panel) and SEM images of the GCN membrane (lower panel) close to the left and to the right reactor sections. (e–g) Reprinted and adapted from ref. 82.

Simultaneous sublimation and polycondensation of melamine in the neighbourhood of an AAO membrane in a closed reactor at 500 °C resulted in the uniform deposition of a GCN layer on the inner walls of AAO channels^87,88^ (Fig. 1a and b).

After the dissolution of the AAO template, macroscopic close-packed arrays of GCNTs can be produced with a thickness of around 60 μm and area of about 0.2 cm^2^ preserving mechanical stability and flexibility (Fig. 1c). The thickness of the GCN layer on the AAO walls and, therefore, the inner diameter of the channels of the final GCN membrane, depends on the amount of melamine introduced into the reactor and can be varied in a highly controlled way between 10 and 100 nm (Fig. 1d).^87^

A vapor-deposition polycondensation process can also be applied to produce asymmetric GCNT membranes using conventional symmetric AAO membranes as a template.^82^ In this modification, the AAO membrane divides the reaction chamber into two sections (Fig. 1e) and the products of melamine polycondensation are carried at 500 °C through the channels of the AAO membrane by a nitrogen flow. The concentration of precursor vapors in the reactor section before the AAO membrane (*C*_H_) is higher than in the section behind the AAO membrane (*C*_L_) due to the slow diffusion of the precursor through the AAO channels. This gradient of precursor vapor pressure results in the formation of asymmetric GCN walls (Fig. 1f). After the dissolution of the AAO template, a GCNT membrane forms with the diameter of the channel increasing from 15–20 nm at the side contacting the left reactor section to 70–80 nm on the side contacting the right reactor section (Fig. 1g).^82^

The GCNTs can be formed inside the AAO membrane channels *via* the thermal decomposition of a precursor formed by reacting ethylenediamine and CCl_4_ at 90 °C and introduced into the pores of the alumina template by an ultrasound treatment.^89^ However, the GCN tubes produced by this method revealed a low adhesion to each other and only an unstructured mixture of separate GCN tubes can be obtained after the dissolution of the AAO template.^89^

A natural clay – halloysite, which has an inherently nanotubular structure was also applied as a template to form porous GCN tubes.^90^ The halloysite was subjected to a pretreatment with HCl to clean the inner pores and heated in a closed reactor with melamine. The latter was sublimed and condensed into GCN on the surface of halloysite tubes, which were then etched by HF resulting in a tubular product with a specific surface area of 86 m^2^ g^−1^ as compared to 6 m^2^ g^−1^ for the bulk GCN produced without any template.^90^

#### “Soft” templates

Apart from AAO membranes and zeolites, which are typically considered as “hard” templates, the conventional techniques of “soft” templating with polymeric globules and micelles^13,17,78^ can be applied to the synthesis of tubular GCN. Here we also discuss the formation of GCNTs templated by metal chloride crystals in eutectic mixtures, which then disappear spontaneously as the temperature is increased over the melting point of the eutectic.

In particular, amphiphilic copolymer Pluronic F127 (HO–(CH_2_CH_2_O)_*n*_–(CH_2_CH_2_CH_2_O)_*m*_–H) was used to form GCNTs by annealing mixtures of this polymer with urea and thiourea.^91^ The procedure yields GCNTs with a diameter of 300–350 nm and relatively thin walls of 20–40 nm. This reference, however, provides the morphological characterization on the samples prepared with only one ratio of components and no details on a possible mechanism of tube formation. Therefore, no definite conclusions on the relationship between the shape of the original polymer micelle and the shape of final GCNT can be drawn. GCNTs co-doped with Na and S were synthesized by a similar protocol with Pluronic F127, urea, as well as thiourea and NaHCO_3_ and a source of sulfur and sodium, respectively.^92^

**Fig. 2:**
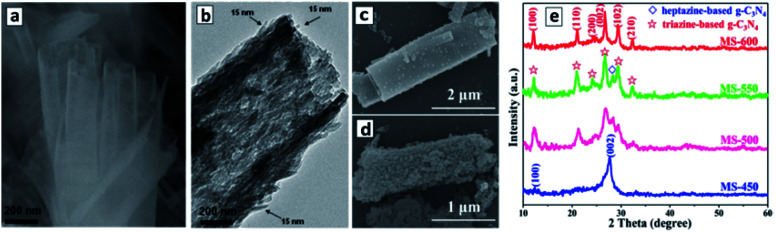
(a–d) SEM (a, c and d) and TEM (b) images of tetragonal GCNTs produced in a LiCl–KCl–NaCl eutectic at 420 °C (a), 500 °C (c), and 600 °C (d). (e) XRD patterns of the products of the melamine polycondensation in the eutectic at different temperatures (450–600 °C). Reprinted with permissions from ref. 94 (a and b) and ref. 96 (c–e). Copyright (2013) The Royal Society of Chemistry (a and b) and (2019) Elsevier (c–e).

In the synthesis of intercalated GCN compounds using eutectic salt mixtures, in particular, LiCl–KCl–NaCl, the cubic salt crystals play the role of templates inducing the formation of tubular GCNs.^93–100^ A typical case of the melamine polycondensation at 550 °C in the eutectic mixture of LiCl × H_2_O–KCl–NaCl (weight ratio of 1 : 1 : 1) yields GCNTs with a 200 nm tetragonal cross section and a length of about 2 μm (Fig. 2a).^94,98,100^ The GCNT walls are composed of agglomerated nanoplates about 100 nm in length and 15 nm thick (Fig. 2b).

The thermogravimetric analysis showed that the polycondensation of melamine starts at a lower temperature (330 °C) than the melting point of the eutectic mixture (355 °C). Inspection of the GCNTs by infrared (FTIR) and X-ray photoelectron spectroscopy (XPS) revealed the presence of cyano-groups C–CN, which were assumed to form as a result of proton subtraction from C–NH_2_ groups by Cl^−^ anions from the eutectic crystals.^94,98^ This observation indicates that the polycondensation of melamine occurs directly on the surface and with the participation of metal chloride crystals. This conclusion was further corroborated by the fact that no GCNTs formed in the eutectic mixture containing GCN instead of melamine. The metal chloride crystals have a cubic form and act as a template determining the shape of future GCNTs even after the crystals are melted at temperatures above 355 °C and the melem is further condensed into GCN at 420 °C.^94^

This approach can be further modified to produce doped GCNTs and to eliminate intercalated ions to increase the specific surface areas of the final product.^95^ In particular, the addition of 1-butyl-3-methylimidazolium hexafluorophosphate along with melamine to the LiCl–KCl–NaCl allows phosphorus as dopant to be introduced instead of nitrogen in some of the NC_3_ positions of the polyheptazine sheets.^95^ Modification of the eutectic with CuCl_2_ allows doping the final GCNTs with copper(ii) ions.^97^

The metal chloride eutectic synthesis strategy was further developed to produce combined polyheptazine/polytriazine homojunction tubes simply by tuning the temperature of the polycondensation in the molten salt mixtures.^96^ It was found that an increase in the polycondensation of melamine in the LiCl–KCl 1 : 1 eutectic at 450 °C results in polyheptazine-based GCNTs with XRD patterns identical to those of bulk GCN. At the same time, the products of polycondensation at 600 °C consist exclusively of the polytriazine imide (PTI) phase, while mixtures of polyheptazine and polytriazine form at intermediate temperatures between 450 °C and 600 °C (Fig. 2c).^96^ No GCN-based products were detected at temperatures below 450 °C and no products at all can be obtained at temperatures higher than 650 °C,^96^ thus clearly defining the temperature range of interest for the synthesis of different carbon nitride allotropes. The PTI phase was observed to be deposited on GCNTs as discrete 20–30 nm particles, their number increasing with the polycondensation temperature till the entire GCN is converted into PTI at 600 °C, the products still retaining a tube-like morphology (Fig. 2d). The PTI is believed to be produced by the thermal decomposition of polyheptazine and recrystallization as a separate phase assisted by the molten salt environment.^96^

Melamine formaldehyde resin was probed as a template for the synthesis of tubular GCN by the co-polycondensation with urea.^101^ The resin molecules have a shape of coiled springs with melamine residual groups abundantly available for the growth of a polyheptazine network. As a result, the “coiled-spring” shape is translated from the resin-template to the final GCN resulting in wound tubular superstructures with a diameter of around 50 nm and a wall thickness of 10 nm.^101^

An original “self-templating” synthesis of GCNTs exploited the technique of unidirectional freezing of aqueous urea solution.^102^ Due to a temperature gradient from the reactor bottom cooled with liquid nitrogen the urea solution was frozed in the form of microrods, which converted after water sublimation into arrays of urea microrods. The calcination of these arrays produced mesoporous GCNTs with wall pores of 10–20 nm and diameters of hundreds of nanometers.^102^

### 2.2 Synthesis of GCNTs with supramolecular precursor assemblies

**Fig. 3:**
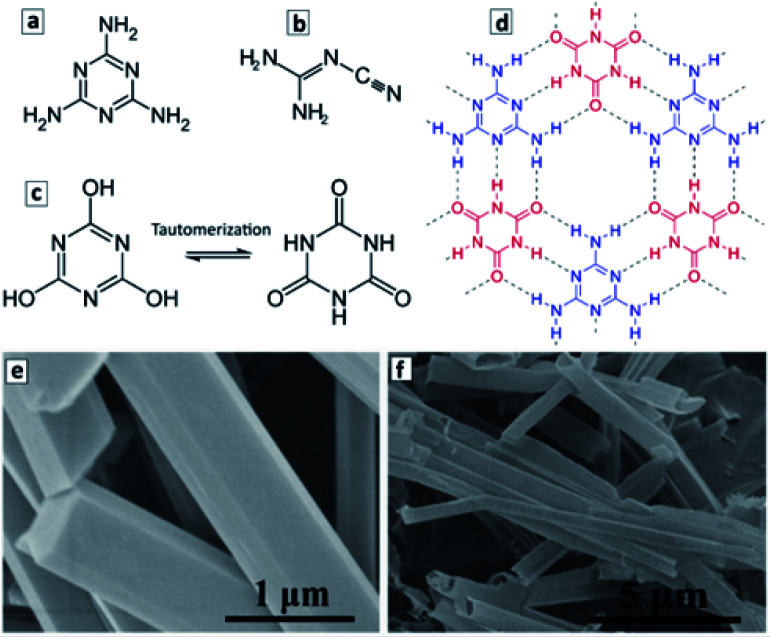
(a–d) Structural formula of melamine (a), dicyandiamide (b), cyanuric acid (two tautomeric forms in (c)), and a fragment of the adduct of melamine and cyanuric acid (d). Structural formulae reprinted from Wikipedia. (e and f) SEM images of M + CA adduct microrods (e) and GCN microtubes produced by the calcination of the adduct microrods at 550 °C (f). Reprinted with permissions from ref. 103. Copyright (2019) American Chemical Society.

The molecular precursors used for the synthesis of GCN are typically rich in nitrogen and have multiple functional groups, such as C–NH_2_ groups in melamine (Fig. 3a) and dicyandiamide (Fig. 3b), or C–OH groups in cyanuric acid (Fig. 3c). When two or more precursors are combined they can evolve into complex ordered structures due to the formation of hydrogen bonds as exemplified in Fig. 3d for melamine and cyanuric acid (CA). This opens the unique opportunity of supramolecular assembling of precursors into 3-dimensional ordered structures, such as polyhedral rods or ribbons and translating such geometry to the final GCN materials produced *via* the calcination of such supramolecular objects.^103–107^

Fig. 3c and d illustrate the formation of a 2D supramolecular network of melamine (M) and tautomerized CA molecules *via* hydrogen bonding between the CNH_2_ groups of melamine and carbonyl groups of the tautomerized form of CA. The ππ-stacking of such 2D supramolecular assemblies gives rise to 3D objects with a well-defined morphology such as microrods with a hexagonal cross section. Typically, such microrods (Fig. 3e) are produced under the conditions of a hydrothermal treatment (HTT) at 150–200 °C in supercritical water.^103^ The calcination of such microrods at 500–600 °C results in the formation of hollow microtubes with the walls composed of GCN (Fig. 3f).

The synthesis of GCNTs through supramolecular assemblies is very flexible and allows the properties of the final product to be tuned both in the stage of the assembly formation and in the stage of the final calcination. The first approach can be exemplified by the introduction of a third component – caffeine into the supramolecular M + CA assembly.^104^ It is supposed that caffeine builds into the assembly as an “edge” molecule, which forces the methyl groups of caffeine to move out of the molecular plane and to twist the entire supramolecular assembly during the polycondensation thus favoring to the formation of tubular products. Additionally, the introduction of caffeine results in an increase of subbandgap defect-related absorption of the final GCNTs proportionally to the caffeine content in the original assembly.^104^

The structure of the supramolecular assemblies can also be affected by introducing additional templates – polyionic liquids, which can form micelles and affect the morphology of the supramolecular M + CA aggregates.^108^ In comparison to the non-templated rod-like M + CA assemblies that exhibited a diameter range of 10–50 μm, the aggregates produced by the HTT of ternary mixtures of M with CA and poly(1-butyl-3-vinylimidazolium bromide) appeared to be much smaller with a diameter of about 1 μm, which was translated to the final GCNTs.^108^

**Fig. 4:**
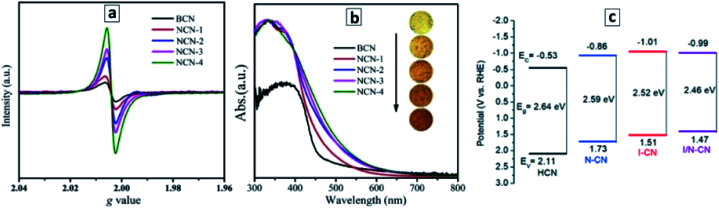
(a and b) Room-temperature EPR spectra (a) and absorbance spectra (b) of bulk GCN (BCN) and GCNT samples produced at different durations of the post-synthesis thermal treatment at 550 °C – 15 min (1), 30 min (2), 45 min (3), and 60 min (4). Reprinted and adapted with permissions from ref. 105. (c) Band levels of GCN tubes produced with no acids present (HCN), with nitric acid (N–CN), with iodic acid (I–CN), with both acids (I/N–CN). Reprinted and adapted with permissions from ref. 109. Copyright (2019) The Royal Society of Chemistry.

A good example of the second approach, the post-synthesis modification of the properties of GCNTs, is the introduction of nitrogen vacancies into the GCNTs produced by an additional thermal treatment of GCNTs synthesized from a M–CA assembly.^105^ A treatment of GCNTs at 550 °C in ambient atmosphere results in a partial disruption of the polyheptazine network and elimination of the tertiary nitrogen atoms, generating in this way nitrogen vacancies with broken bonds than can be observed by electron paramagnetic resonance (EPR) spectroscopy.^105^ As the treatment duration is increased from 15 to 60 min, the population of the N vacancies grows resulting in a higher EPR signal (Fig. 4a) as well as in a narrowing of the bandgap from 2.72 eV to 2.42 eV (Fig. 4b).^105^

Similarly, the introduction of strongly oxidizing nitric and iodic acids during the polycondensation of M + CA assemblies induces the formation of carbon and nitrogen vacancies as well as a partial conversion of the terminal CNH_2_ groups of the GCN sheets into cyano-groups.^109^ Both factors result in a narrowing of the bandgap of GCNTs from 2.64 eV to 2.46 eV as well as in a shift of the CB potential to more negative values (Fig. 4c).

The formation of GCNTs with porous walls from a mixture of melamine and urea was also found to proceed *via* the stage of the supramolecular M + CA assembly with cyanuric acid produced through the *in situ* polycondensation of urea.^107^ The tubes prepared directly from M + CA assemblies had a length of 200–800 nm, a wall thickness of 15 nm, and a diameter of 150 nm, while the polycondensation of melamine and urea mixtures resulted in larger tubular formation with a length of 2–3 μm, a wall thickness of 40 nm, and a diameter of around 250 nm. The evolution of gases (NH_3_, CO_2_, H_2_O) during the polycondensation of the M + CA precursor resulted in 5–20 nm mesopores in the GCNT walls.^107^

The supramolecular precursors for GCNTs can also be formed using melamine as a sole starting molecular material. In this approach, melamine is subjected to a HTT in aqueous solutions and is partially converted to cyanuric acid, which then forms supramolecular assemblies with residual melamine. The depth of melamine conversion and the morphology of the supramolecular precursor can be affected by changing the solvent properties (pure water,^110–112^ strong alkalis^113^ or acids^114–116^) as well as the duration and temperature of the HTT.^110,111^

**Fig. 5:**
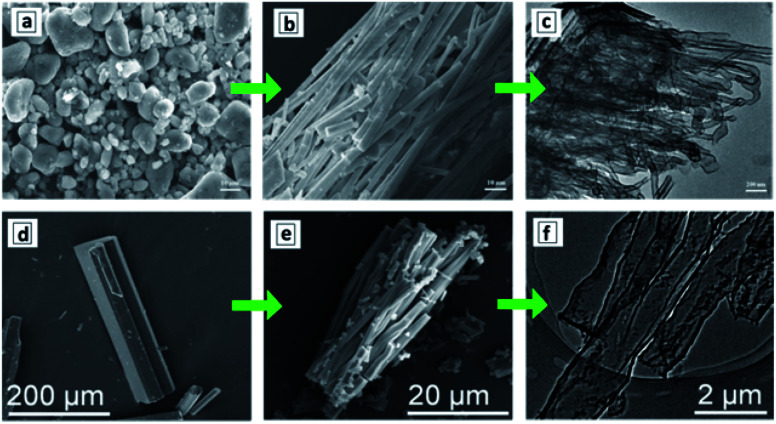
SEM (a, b, d and e) and TEM (c and f) images of melamine (a), M + CA assemblies (b, d and e) and GCNTs (c and f). Reprinted and adapted with permissions from ref. 110 (a–c) and ref. 114 (d–f). Copyright (2018) Elsevier (a–c) and American Chemical Society (d–f).

In the simplest case, the HTT of melamine in DI water produces well-resolved and uniformly shaped tubular supramolecular aggregates (Fig. 5a and b), which transform into the GCNTs upon calcination at 550–600 °C.^110,117^ The GCNTs produced in such conditions reveal a length of several microns, a diameter of 30–60 nm (Fig. 5c),^110^ and a bandgap of 2.75 eV close to the *E*_g_ of bulk GCN produced without the HTT stage.^110^ However, in comparison to bulk GCN, the GCNTs reveal stronger and broadened EPR signals indicating the presence of a higher density of nitrogen vacancies, which impart the GCNTs with a much higher photochemical activity as discussed in the next sections.

It was proposed to modify this protocol by performing the HTT of melamine in aqueous solutions containing different amounts of NaCl.^118^ This method yields 20–100 μm microtubes with a tetragonal 2–5 μm cross-section filled with layered matter, which is described by the authors as micrometer-long close-packed GCN nanowires.

Multiple variations in the synthesis protocols of GCNTs reported in different papers makes it rather difficult to evaluate the influence of different conditions of the melamine HTT on the properties of final GCNTs. Still, some trends can be clearly observed, in particular, an increase in the diameter of GCNTs with an increase in the treatment duration (from 30–60 nm at 8–16 h (ref. 110) to 1–2 μm for 24 h treatment^112^).

The hydrothermal treatment of melamine in the presence of concentrated NaOH produces supramolecular rod-like aggregates with a diameter of 1–3 μm converting upon calcination into sodium-doped GCNTs.^113^ The tubular GCN preserves the morphology of the original supramolecular precursor revealing a diameter of 1–3 μm and a length of 10–40 μm.^113^ The NaOH concentration during the HTT determines the sodium content in the final GCNTs, but almost does not affect their diameter and length allowing the mode of doping (by NaOH concentration) and the tube morphology (by HTT duration and temperature) to be tuned separately. In a similar way, K and Li-doped GCNTs can be produced.^113^

The HTT of melamine in the presence of H_3_PO_4_ was also found to yield rod-like supramolecular aggregates of melamine with CA (Fig. 5d) converting upon calcination to GCNTs with a diameter of about 80 μm and a length of up to 350 μm.^114–116^ A second relatively short (8 h) HTT of the supramolecular M + CA microrods in aqueous solutions of concentrated KOH was reported to result in the precursor reconstruction into 15–20 μm bundles of much smaller 1–3 μm rods (Fig. 5e) yielding upon calcination GCNTs with a diameter of around 1 μm (Fig. 5f).^114^ We should note that the morphology and composition of the final GCNTs produced by this two-stage HTT and the direct one-stage treatment with NaOH are roughly the same indicating the same formation mechanism of supramolecular assemblies in both cases. The doping of GCNTs with phosphorus in NC_3_ node positions of the heptazine heterocycles was reported to occur irrespectively of the phosphorus source used in the synthesis, including sodium pyrophosphate, ammonium phosphate, sodium hypophosphite, and sodium phosphite.^119^

A detailed study of the supramolecular precursor forming at HTT of melamine with phosphorous acid^120^ showed that it crystallizes as hexagonal-shaped microrods with a length of 300–500 μm and a diameter of 600–700 μm. Each rod is a ππ-bound stack of nanometer-thin sheets of supramolecular assemblies of melamine and CA, the latter forming *via* the *in situ* melamine hydrolysis during the HTT.^120^ Upon calcination of the supramolecular precursor, a partial substitution of nitrogen atoms in heptazine NC_3_ positions with P atoms was observed resulting in a narrowing of the bandgap to 2.55 eV (*E*_g_ = 2.67 eV for bulk GCN produced without H_3_PO_4_).^120^

**Fig. 6:**
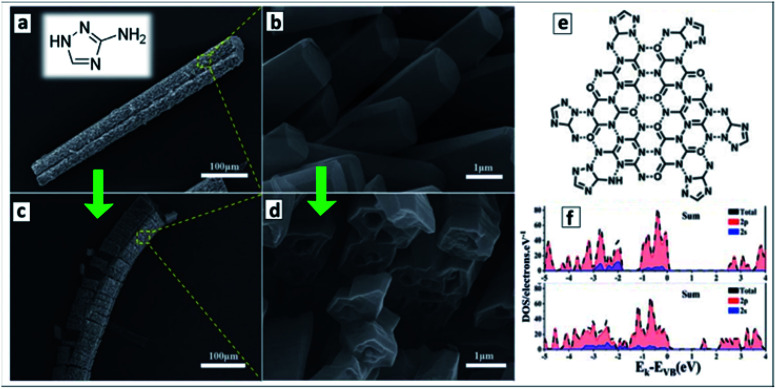
(a–d) SEM images of supramolecular M + CA + AT precursor (a and c) and final GCNTs (b and d); (e) assumed structure of supramolecular M + CA + AT assembly; inset in (a): molecular structure of 3-amino-1,2,4-triazole (reprinted from Wikipedia). (f) Density of states of GCN (upper panel) and triazole-containing GCNTs (lower panel). Reprinted and adapted with permissions from ref. 121. Copyright (2020) The Royal Society of Chemistry.

The hydrothermal treatment of melamine with 3-amino-1,2,4-triazole (AT) was found to result in a ternary supramolecular M + CA + AT precursor crystallizing as microrods covered with arrays of nanorods (Fig. 6a and b).^121^ The calcination of the rod-like precursor at 550 °C in N_2_ atmosphere converts it to a “nanotubes-on-a-microtube” formation (Fig. 6c and d) reflecting the morphology of the original supramolecular assembly. A feasible structure of the precursor supramolecular assembly was proposed based on the results of X-ray diffraction (XRD), FTIR and nuclear magnetic resonance (NMR) spectroscopies (Fig. 6e). Inspection of the final GCNTs reveals the presence of residual triazole heterocycles bound to the polyheptazine carcass of the tubes, which contributes to the narrowing of the bandgap of the GCNTs (*E*_g_ = 2.45 eV) as compared to that of the conventional bulk GCN (*E*_g_ = 2.73 eV).^121^ DFT calculations of model terminal fragments of triazole-doped GCN sheets showed that the “alien” triazole heterocycle introduces N-related states into bandgap of GCN close to the CB edge (Fig. 6f).^121^

Introduction of additional carbo- and heterocycles is currently one of recognized methods of affecting the bandgap, CB/VB levels, and spectral sensitivity range of bulk and nanosheet GCN materials.^29^ The present example shows that in the case of GCNTs this approach can be combined with the design of the supramolecular precursors, the same heterocycle playing the role of both structure-directing agent of the precursor and dopant of the final GCNTs.

The HTT of melamine mixed with hydroxylammonium chloride H_2_N–NH_2_ × HCl results in the formation of a supramolecular assembly differing in the lattice parameters from both the original melamine and bulk GCN and crystallizing in the form of microrods with a length of several microns and a diameter of around 200 nm.^122^

Calcination of such aggregates in an ammonia atmosphere yields 2–3 μm GCNTs with a diameter of 30–60 nm and a lower C/N atomic ratio as compared to that of bulk GCN due to the abundant presence of surface amino-groups.^122^ The supramolecular assemblies formed from melamine and hydroxylammonium sulfate under the HTT have the shape of thin ribbons with a length of up to 20 μm, which convert upon calcination into GCNTs with a mean diameter of 10 nm.^123^ No mechanistic details of this conversion are provided in ref. 123, but the scrolling of ribbons into nanotubes can be apparently considered as the most probable route to GCNTs in this system.

**Fig. 7:**
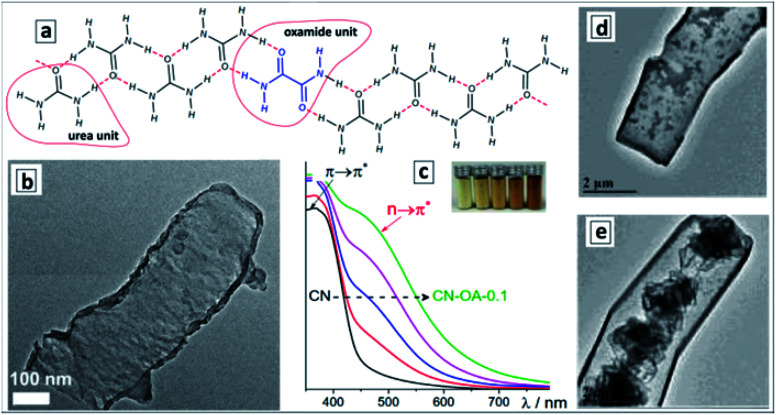
(a) Proposed structure of urea-oxamide assembly; (b) TEM image of a GCNT; (c) absorption spectra of GCN samples produced from urea-oxamide (OA) mixtures with a mass fraction of OA varying from 0 to 0.1. (a–c) Reprinted and adapted from ref. 124 – published by The Royal Society of Chemistry. (d and e) TEM images of GCNTs produced from melamine/urea mixtures with calcination in air (d) and in N_2_ atmosphere (e). Reprinted and adapted with permissions from ref. 125. Copyright (2019) The Royal Society of Chemistry.

Formation of supramolecular assemblies between urea and oxamide molecules takes place spontaneously at the evaporation of their mixed solutions allowing GCNTs to be obtained by the direct thermal decomposition of the assembly without intermediate hydrothermal treatments.^124^Fig. 7a shows the proposed structure of the ribbon-like supramolecular assembly of urea and oxamide units converting upon polycondensation into a polyheptazine network. The GCNT formation (Fig. 7b) was observed at the mass fraction of oxamide higher than 0.05. As the oxamide content was gradually increased, the bandgap of the final GCN narrowed and the absorption band edge shifted from about 470 nm to 600–650 nm without considerable changes in the band structure (Fig. 7c).

This phenomenon was assumed not to stem from the rise of defect-related subbandgap absorbance but from a contribution of n–π* transitions with the participation of lone electron pairs of nitrogen atoms on the edges of the polyheptazine ribbons.^124^ The authors argue that the shape of GCNTs favors the distortion of the planar polyheptazine structure increasing the probability of such n–π* transitions, which are forbidden by symmetry for conventional bulk GCN. This example clearly shows the feasibility of affecting the electronic and optical properties of carbon nitride sheets by tailoring them into tubular formations.

The calcination of supramolecular assemblies produced by the hydrothermal treatment of melamine/urea mixtures results in GCNTs with a diameter of 1–2 μm and a length of about 20 μm.^125^ The report provides no details on the structure and composition of the precursor assemblies, which can be formed both by melamine/urea and the products of the hydrolysis of both these compounds. A pronounced effect of the calcination atmosphere on the structure of final GCNTs was found, the tubes prepared in air showed hollow internal channels (Fig. 7d), while the tubes synthesized under N_2_ atmosphere contained crumpled CN sheets inside the channels (Fig. 7e). The latter structure can be very favorable for applications in sorption and catalysis providing a much higher surface area.

The formation of supramolecular M + CA assemblies during the HTT can be achieved using only DCDA as a sole precursor.^126^ Based on a combination of FTIR, XPS, and solid-state NMR results, the authors suggested a feasible mechanism of this process, including (i) the cyclization of DCDA into tautomerized CA with the subtraction of ammonia and guanidine (HN=C(NH_2_)_2_) molecules and (ii) the conversion of CA into melamine at the expense of ammonia released from DCDA as well as from the *in situ* hydrolysis of guanidine.^126^

A hydrothermal treatment of melamine or DCDA in tetrachloromethane CCl_4_ yields a black powdered product, which converts to a mixture of tubes and belts upon calcination.^127^ The belt width ranges from 100 nm to 3 μm with a thickness of 5–50 nm, while the tubes are characterized by a relatively small inner diameter of 5–20 nm as compared to the outer diameter of 70–200 nm and a length of several micrometers.^127^

**Fig. 8:**
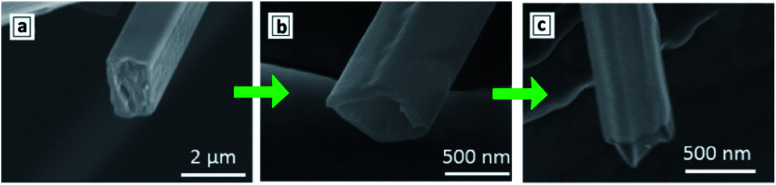
SEM images of a tetragonal prism produced by polycondensation of melamine in a CCl_4_ flow before treatment (a), after dissolution of inner matter with water (b), and after calcination (c). Reprinted with permissions from ref. 128. Copyright (2019) Elsevier.

The polycondensation of melamine in a CCl_4_ flow was reported to result in hollow tetragonal prisms with a length of hundreds of micrometers and a wall thickness of around 50 nm.^128^ It is assumed that chlorine atoms forming at the decomposition of CCl_4_ bind to sublimed melamine molecules *via* hydrogen bonds, the associate moving with the CCl_4_ flow and condensing further in the reactor in the form of elongated tetragonal prisms (Fig. 8a).

As the reaction proceeds, the polycondensation degree of the external walls increases, the chlorine desorbing in the form of NH_4_Cl. Such prisms still contain low-condensed chlorine-rich intermediates in the interior of the tube, which can be removed by dissolution (Fig. 8b), and the final tetragonal GCNTs produced after calcination at 500 °C (Fig. 8c).^128^

### 2.3 Synthesis of GCNTs through scrolling of nano-/microsheets

As the structure of bulk GCN is very similar to that of graphite, the most straightforward way to produce tubes can be by exfoliating the layered bulk material into single layers and force them to coil and form the tubes. Numerous studies using ultrasound treatment of bulk GCN in various solvents showed the exfoliation to yield mostly few-layer GCN nanosheets comprising 5–10 single polyheptazine layers,^16,21,53,54^ which can serve as a precursor for the production of GCNTs through scrolling. The rolling-up of the nanosheets can be achieved by various approaches including HTT,^129^ shock freezing,^130^ and anti-solvent addition.^131^

The hydrothermal treatment, most probably, results in a “softening” of the GCN sheets and an increase in their flexibility, which then allows the sheet to minimize its contact area with the dispersive medium by scrolling into a tube. The method appears to be versatile, as it allows nanosheets of a different thickness to be processed as well as additional components to be introduced, which undergo chemical conversions during the treatment. The *in situ* reduction of metal compounds during HTT results in an uniform decoration of inner and outer GCNT walls with nanocrystalline Pt,^132^ Ag,^133^ Rh,^134^ or with polyoxymetallate species.^129^

**Fig. 9:**
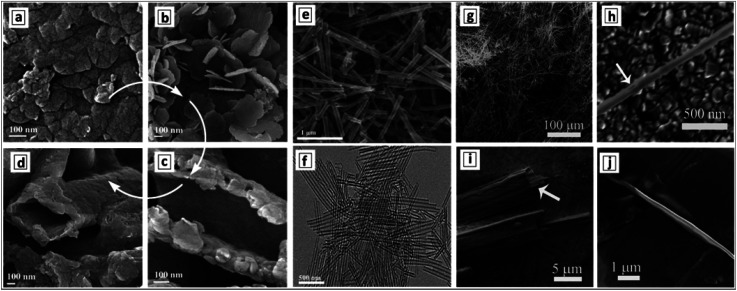
SEM images of bulk GCN (a), GCN nanosheets (b), GCN tubes (d–h), and products of incomplete rolling of the sheet precursors (c, i and j). Reprinted with permissions from ref. 129 (a–d), ref. 143 (e and f), and ref. 142 (g–j). Copyright (2014) Elsevier (a–d), (2019) American Chemical Society (e and f), and (2012) The Royal Society of Chemistry (g–j).

The hydrothermal treatment of GCN sheets, produced by the ultrasound-assisted peeling of bulk GCN, results in the formation of GCNTs with an outer diameter of around 100 nm and a wall thickness of 10 nm.^129^ The examination of the products of ultrasound and hydrothermal treatments of bulk GCN allows the course of conversion of bulky GCN aggregates (Fig. 9a) into individual nanosheets (Fig. 9b), their gradual scrolling (Fig. 9c), and formation of final tubes (Fig. 9d) to be tracked.

The formation of GCNTs can be achieved by applying a second ultrasound treatment to suspensions of protonated GCN nanosheets produced in the first sonication from bulk GCN without additional calcination steps.^135^

A rapid drop in temperature from 350 °C to below zero resulted in coiling of the hot GCN nanosheets exfoliated by the ultrasound treatment of bulk GCN and the formation of GCNTs.^130,136^ The tubes showed a broad scatter of diameters indicating a low level of morphological control in this approach. It was assumed^136^ that the external layers of the hot GCNT nanosheets cool much faster in the below-zero water than the inner layers generating thermal stress, resulting in a plastic deformation, and, finally, coiling of the nanosheets into GCNTs. Water is characterized by a relatively low thermal conductivity (more than 600 times lower than copper) allowing the hot nanosheets to be maintained in a state of thermal stress long enough for this morphological transformation to occur.^136^

Carbon dot-decorated GCNTs were reported to form by calcination of a precursor mixture of graphitic carbon dots with urea produced by freeze-drying of an aqueous precursor solution in liquid nitrogen.^137^ The authors assumed amide bonding to occur between urea and carbon dots resulting in tubular aggregates with walls reinforced by the incorporated carbon dots. This method, however, provides only limited means of control over the morphology of the tubes, because the GCNTs collapse at a higher content of carbon dots and transform into graphene-like crumpled sheets.^137^

The freeze-drying of a precursor produced by a short HTT (180 °C for 4 h) of aqueous DCDA solution with a following calcination at 600 °C was reported to yield tubular GCN formations.^138^ The authors assumed the tubes to form during the annealing due to nanosheet scrolling along the flow of releasing ammonia. However, as the HTT was applied to prepare the precursor, which can result in the hydrolysis and partial condensation of DCDA, the formation of supramolecular assemblies cannot be excluded followed by their conversion into GCNTs upon calcination as discussed in detail in the previous section.

A similar scrolling mechanism was suggested for the formation of phosphorus-doped GCNTs by the calcination of mixtures of melamine and sodium hypophosphite monohydrate (NaH_2_PO_2_ × H_2_O).^139^ It is assumed that phosphine is released during the calcination, forcing the GCN nanosheets to coil into tubes and creating mesopores in the tube walls. The GCNTs produced by this method are characterized by a diameter of around 200 nm and a wall thickness of 20–50 nm.^139^ The GCNTs reveal a specific surface area of 13.4 m^2^ g^−1^ as compared to 3 m^2^ g^−1^ for bulk GCN and a wall mesopore size of 10 nm. Such morphology can be expected to be very favorable for photocatalytic reactions allowing reactants to be efficiently collected in the pore structure and trap the incoming light due to the tubular shape of the photocatalyst. A similar approach was applied to synthesize barium- and phosphorus-co-doped GCNTs starting from melamine and hypophosphorous acid.^140^

A scrolling event stimulated by an ammonia flow through the melamine upon thermal condensation was suggested to be responsible for the formation of GCNTs during the calcination of close-packed melamine.^141^ The melamine packing was achieved by shaking the crucible with a vibrator prior to the calcination, the latter yielding GCNT bunches with an inner tube diameter of 18 nm and a wall thickness of 15 nm.^141^

Formation of very long and uniform GCNTs with an aspect ratio on the order of 10^5^ was reported^142^ to occur by calcination of a nanoribbon precursor, the latter forming in ethylene glycol by the interaction between melamine and nitric acid. The XRD pattern of the precursor differs from those of both melamine and GCNTs, revealing it to be layered and hierarchical, however, no more details on the structure of this supramolecular formation were reported. The GCNTs are up to several millimeter long and have an outer diameter of 300–500 nm (Fig. 9g and h) and a wall thickness of around 20 nm.^142^ This morphology makes the GCNTs readily processable for the fabrication of single-tube-based light-sensitive devices such as photodetectors. A detailed inspection of numerous GCNTs revealed the presence of incompletely scrolled nanoribbons (Fig. 9i and j) indicating the scrolling as the most plausible mechanism of the GCNT formation.

A similar procedure involving the interaction of melamine and concentrated HNO_3_ in ethylene glycol was reported to yield much shorter 1.3 μm uniformly shaped GCNTs with a diameter of around 100 nm (Fig. 9e and f).^143^ The tubes were decorated by Au–Pd NCs forming simultaneously with the tubes in the stage of calcination. Differences in the tube morphology between the products reported in ref. 142 and 143 can arise from a different concentration of melamine in the original solution (higher in ref. 142) and calcination temperature (higher in ref. 143) indicating possible ways of controlling the length of final GCNTs in these syntheses.

The calcination of a mixture of melamine and salicylic acid was found to result in the formation of GCN nanosheets, which then spontaneously scrolled into 4–5 μm long tubes with a diameter of around 60 nm and porous 10 nm thick walls.^144^ It was assumed that salicylic acid forms complexes with amino-groups of melamine affecting the polycondensation dynamics and precluding the formation of multi-layer stacked bulk GCN structure. As a result, predominantly few-layer sheets were formed, which tended to coil into tubes to reduce the surface energy.^144^

In cases of total exfoliation of bulk GCN to single-layer carbon nitride or even smaller fragments of the polyheptazine network such as melem hydrate,^131^ the formation of tubes can be initiated by addition of an “anti-solvent”. The latter results in the destabilization of the colloidal single-layer carbon nitride and coiling of the ultra-thin sheets trying to minimize the contact with the anti-solvent. Such an approach was realized by the exfoliation of bulk GCN in concentrated H_2_SO_4_.^131^ The exfoliation of GCN into single layer melem hydrate was initiated by a rapid heating at water addition to the concentrated acid resulting in transparent and stable colloidal solutions. Subsequent addition of methanol to the melem colloids yielded tubular formations converted into porous GCNTs upon calcination at 550 °C.^131^

## 3 Applications of graphitic carbon nitride tubes

Due to the unique combination of electronic, chemical, and photochemical properties of GCN, the majority of reports on the properties of GCNTs are focused on various light-harvesting applications of such materials, including photocatalytic reactions, light harvesting in the form of capacitors, and, most recently, on light-induced motion of the GCNTs. Other major applications involve the electrochemical activity of various materials attached to GCNT carriers.

Single and multi-layer GCNTs were subject of quite intense studies by computational methods,^66^ including first principles and molecular mechanics simulations aimed at establishing diameter-dependent electronic and optical properties of GCNTs as well as their possible applications, the feasibility of which is still to be verified by experiments, in particular, in the case of hydrogen storage technologies.^61^

### 3.1 Properties and possible applications of GCNTs predicted by computational methods

The first principle calculations of the electronic structure of GCNTs made of scrolled polyheptazine layers showed that the conversion of the polyheptazine sheets into tubes should result in a narrowing of the bandgap and an increase of charge carrier mobility,^145^ both factors favorable for the light-harvesting applications of GCNTs. The calculations also showed that the functionalization of GCNTs with single metal atoms, in particular Pt and Pd, results in a further decrease of the bandgaps and an enhancement of the charge carrier mobility.^145^

Calculations also showed that the preferable configuration of nitrogen atoms depends on the tube diameter and the presence of carbon vacancies.^146^ In particular, pyridine-like N is more favorable with decreasing tube diameter, while graphitic nitrogen is more favorable for larger nanotubes. The introduction of a carbon vacancy makes the pyridine-like configuration of three neighbouring nitrogen atoms more preferable even for larger nanotubes indicating that the tuning of the C/N ratio of GCNTs can strongly affect their electronic and adsorption properties relevant for catalytic and sensing applications.^146^

Doping with metal atoms was also predicted to affect strongly the magnetic properties of GCNTs, the W- and Ti-doped tubes showing ferromagnetic behavior, while Cr-, Mn-, Co-, and Ni-doped tubes are expected to be antiferromagnetic due to the anti-alignment of the magnetic moments between neighboring metal atoms.^147^ This behavior makes GCNTs a promising material for spintronics and hydrogen storage applications.

**Fig. 10:**
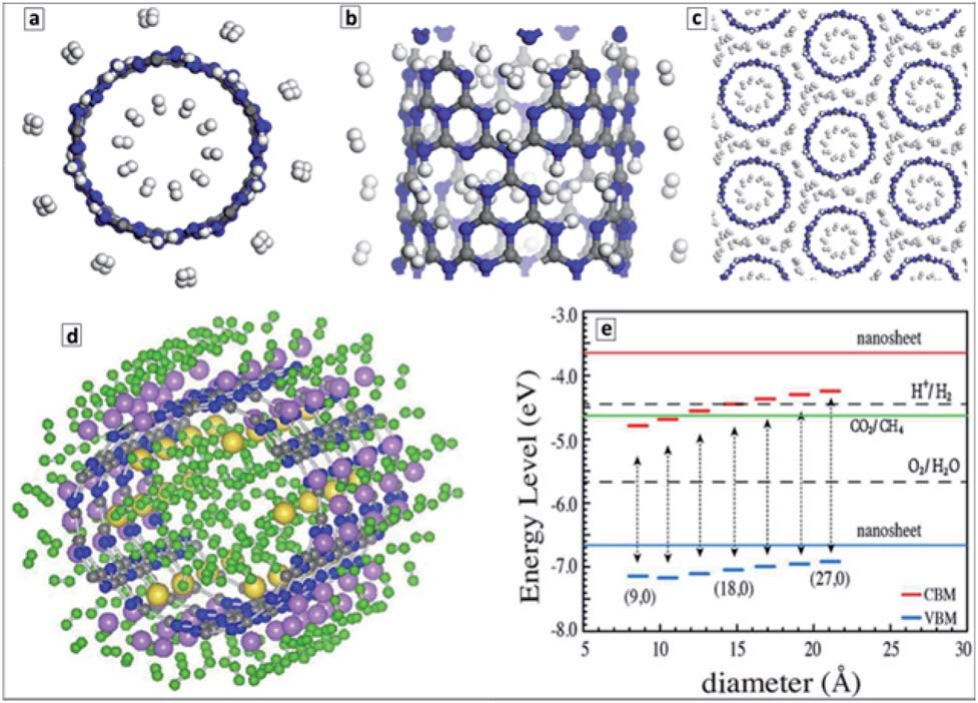
(a) End-on view, and (b) side-view of a representative GCNT structure with adsorbed hydrogen molecules (C-gray, N-blue, H-white); (c) end-on view of a representative structure a GCNT bundle with a hydrogen storage capacity of 5.45 wt%. (d) Optimized atomic structure of (H_2_)_60_/Li_18_/Na_6_/CNT unit (H-green, C-gray, N-blue, Li-pink, Na-yellow); (e) VB and CB level positions for GCNTs of different diameter along with the energy levels of processes of water reduction and oxidation, and CO_2_ reduction to CH_4_. Reprinted with permissions from ref. 148 (a–c), ref. 149 (d), and ref. 152 (e). Copyright (2012) Elsevier (a–d) and (2013) The Royal Society of Chemistry (e).

From the viewpoint of hydrogen storage, the attractivity of GCNTs arises from (i) their porosity allowing easy access of H_2_ to the tube interior void and (ii) the abundance of N=N fragments at the pore edges that can serve as hydrogen adsorption sites as well as be functionalized with single metal atoms with a strong affinity to molecular hydrogen.^148^ Estimations showed that an isolated GCNT can bind up to 4.66 wt% of hydrogen on both sides of the tube (Fig. 10a and b), while for tube bundles the H_2_ adsorption capacity increases to 5.45 wt% due to additional pores between the tubes (Fig. 10c). It is noted that this value is close to the current gravimetric hydrogen storage capacity of pressurized tanks and metal-doped carbon nanotubes. The calculations also revealed the barrier of H_2_ passage through the pores to be relatively low, around 0.54 eV per H_2_, promising a high adsorption/desorption rate at relatively low pressure and temperature.^145^

The hydrogen storage was predicted to increase considerably upon doping of GCNTs with Li^+^ or Na^+^ ions.^149^ These ions can bind 2 (Li) and 4 (Na) H_2_ molecules per ion resulting in a gravimetric hydrogen storage capacity slightly higher than 9 wt%. The average H_2_ adsorption energy was calculated to be in the range of 0.09–0.22 eV per H_2_ molecule, which is suitable for practical hydrogen storage at ambient temperatures.^149^

Fig. 10d illustrates one of the possible stable configurations – (H_2_)_60_/Li_18_/Na_6_/CNT with 36 hydrogen molecules bound to eighteen Li atoms and 24 hydrogen molecules bound to six Na atoms. The volumetric density of such configuration was estimated to be at least 42 kg m^−3^.^149^

It was found that the electric dipole transitions are only allowed along the main axis of the GCNTs,^150^ obviously favoring the spatial separation of photogenerated electrons and holes as compared to conventional GCN without the shape anisotropy.

Density functional theory (DFT) based calculations of the interaction of alkali (Li^+^, Na^+^, K^+^) and alkali earth (Be^2+^, Mg^2+^, Ca^2+^) cations with polyheptazine nanotubes revealed that most of these cations prefer to adsorb on the pores between the heptazine units in the tube walls decreasing the bandgap of the GCNTs.^151^ The cation adsorption can also decrease strongly the electron emission current due to the electron interaction with metals as well as increase the electrical conductance of the GCNTs. This prediction shows high perspectives of such materials as electrochemical sensors for alkali and alkali earth metals with a sensitivity changing in the order: Be^2+^ ≫ Mg^2+^ > Ca^2+^ ≫ Li^+^ ∼ Na^+^ ∼ K^+^.^151^ A similar behavior of GCNTs was predicted toward ammonia molecules, NH_3_ adsorbing preferably on the pores of tube walls and increasing the electrical conductivity of the host.^151^

The properties of single wall CNTs are obviously the most susceptible to the mode of the tube rolling, the nature of terminal functional groups, doping and other modifications. In particular, a combination of molecular dynamics and DFT calculations of single-wall CNTs showed that three possible modes of rolling – armchair, helical, and zigzag – are thermodynamically stable. All armchair CNTs are half-metallic, the helical and zigzag tubes can be tuned from semiconductor to half-metals *via* increasing the tube radius.^153^ The stability of single-layer CNTs with a different chirality comparable to that of unrolled single sheet was explained by a decreased repulsion between unpaired electrons of N atoms in the CNTs.^152,153^

Generally lower bandgaps were reported for CNTs of different geometry as compared to the corrugated polyheptazine sheet.^152–154^ The position of the VB maxima of single-layer CNTs was found to be dependent on the tube diameter and lower in all cases than the corresponding level of the corrugated unrolled nanosheet (Fig. 10e).^152^ In view of these calculations, the CNTs can be expected to reveal a higher photocatalytic activity in oxidation processes than bulk and single-layer carbon nitride. The calculations attest that the band level positions are favorable both for total water splitting and CO_2_ reduction to CH_4_, both expectations corresponding to the reported experimental data. The report also shows that a strong diameter dependence of the electronic properties of CNTs should be expected for very small diameters, *d* < 2 nm.^152,154^

### 3.2 Photocatalytic processes with the participation of GCNTs

As photoactive, visible-light sensitive, and photostable semiconductor, GCN has been broadly studied as a photocatalyst in a variety of redox-processes with a special focus on those constituting the realm of *artificial photosynthesis*, that is, the photocatalytic water splitting to H_2_ and O_2_, or the reduction to hydrogen by electron donors,^16–18,27,30–36,40,155^ CO_2_ conversion to CO and hydrogenated fuels (CH_3_OH, CH_4_),^16,17,27,31,37,156,157^ nitrogen reduction,^19,157^ as well as generation of photocurrent in photoelectrochemical systems.^18,30,31,36,38,49,51^

In the course of gradual progress of the design of low-dimensional GCN-based materials, such as mesoporous and nanolayered GCN, single-layer polyheptazine sheets, GCN NCs, almost each new material, including GCN tubes, has been tested in water splitting and CO_2_ reduction processes and benchmarked against bulk GCN. As the oxidation potential of the photogenerated VB holes in GCN is not high enough to oxidize HO^−^ to HO˙ radicals, the number of photocatalytic degradation processes reported for GCN is much lower than for titanium or zinc oxide, but still many organic compounds can be converted oxidatively over GCN-based materials either by reacting directly with VB holes or by getting peroxidized by O_2_˙^−^, produced from molecular oxygen and CB electrons.^14,16,31^

#### Reductive photocatalytic processes with the participation of GCNTs

This area is dominated by the processes of hydrogen evolution from aqueous solutions of electron donors and CO_2_ reduction with water and other donors. In this subsection we organize the discussion of available reports relative to the strategy, which was used to form the GCNT photocatalyst, that is the templated synthesis, the synthesis through supramolecular assemblies, and scrolling of GCN nanosheets.

The photocatalytic hydrogen evolution is typically reported for the systems comprising powdered GCNT photocatalyst, bare or decorated with 1–3 wt% Pt co-catalyst, dispersed in an aqueous solution of organic electron donors (triethanolamine (TEA), methanol, ethanol, *etc.*), evacuated, and illuminated with a cut-off filter in the range of *λ* > 420 nm. The efficiency of the photocatalytic process is typically expressed by the specific rate of H_2_ formation expressed in mol per hour illumination and per g of photocatalyst as well as by the apparent quantum yield (QY) which is measured with no regard to the light scattering of the photocatalyst and is, therefore, dependent on the illumination wavelength. The photocatalytic reduction of carbon dioxide is studied in similar systems saturated with CO_2_ and containing additional co-catalysts and sacrificial electron donors. A series of possible products can be detected in this process, typically including CO, HCHO, CH_3_OH, CH_4_, and C_2_H_6_.

##### (I) Templated GCNTs

The combination of the tubular morphology and the high surface area of GCNT samples produced using natural clay halloysite as a template resulted in a 14 times higher photocatalytic activity in water reduction as compared to that of bulk GCN.^90^

The GCN material with entangled tubular structure produced using the melamine–formaldehyde resin as a template was demonstrated to be a rather efficient photocatalyst of hydrogen evolution from aqueous TEA solution with an apparent QY of about 19% at 400 nm.^101^

The GCNTs templated by Pluronic F127 were decorated with mixed Pt–Ni NCs^91^ or Pt–Co NCs^92^ and tested as a photocatalyst of the hydrogen evolution from aqueous TEA solutions under illumination with visible light. In the case of Pt–Ni NCs, the H_2_ evolution activity was reported to be extremely sensitive to the composition of metal NCs, being the highest at an equimolar content of both metals and 50 times higher than that for GCNTs without metal co-catalysts.^91^ For the Pt–Co NC-containing Na,S-co-doped tubular photocatalyst an apparent QY of 10.2% at 420 nm was achieved.^92^

**Fig. 11:**
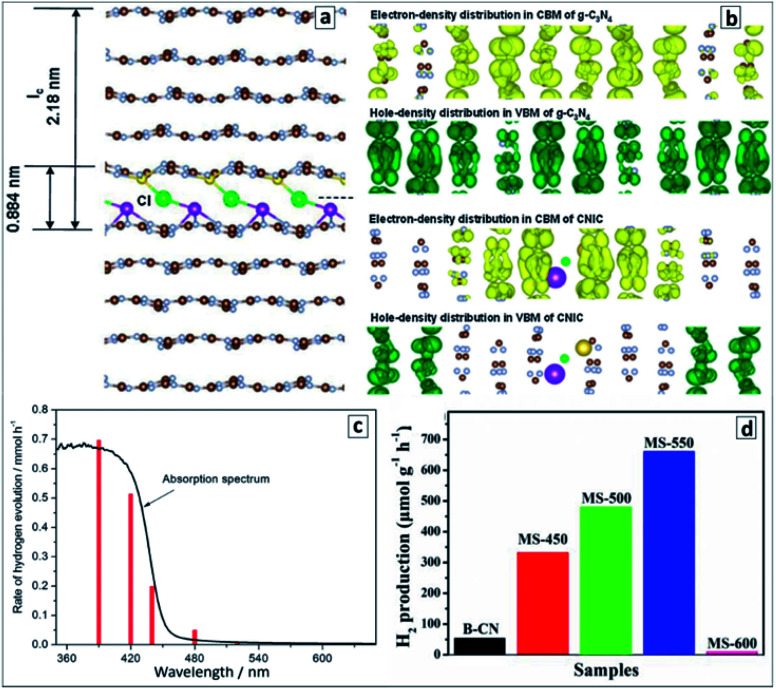
(a) “Stage 5” Na–Cl–K intercalation structure in GCN; (b) charge distributions in CB and VB of pristine (upper panel) and intercalated (lower panel); (c) wavelength-dependent rate of photocatalytic water splitting over the intercalated GCNTs (red bars) and absorption spectrum of the photocatalyst for comparison; (d) rate of the photocatalytic water splitting over GCNT materials produced in molten salt at different calcination temperatures. Reprinted with permissions from ref. 94 (a–c) and ref. 96 (d). Copyright (2013) The Royal Society of Chemistry and (2019) Elsevier.

A very high photocatalytic activity in water splitting using sacrificial donors was reported for the GCNTs produced in molten metal chloride eutectics and intercalated with Li, K, Na, and Cl ions.^94^ The tube formation and ion intercalation occur at different temperatures (420 °C and 500 °C, respectively) allowing for a separation and control over both events. When the temperature is raised over 500 °C intercalation of metal cations occurs into tetragonal GCNTs with the formation of tubular intercalation compounds, which can be described by a brutto-composition C_3_N_4.52_H_3.53_K_0.32_Na_0.12_Li_0.25_Cl_0.08_.^94^ Analysis of the XRD patterns of the intercalates with this brutto-composition revealed it to be a “stage 5” intercalation compound,^94,158^ with each fifth gallery expanded and occupied by the intercalating species (Fig. 11a). This compound exhibited around 35 times higher photocatalytic activity in hydrogen evolution from aqueous TEA solutions in the presence of a Pt co-catalyst as compared with bulk GCN.^94^ The hydrogen production efficiency at different wavelengths follows closely the absorption spectrum of the intercalated GCNTs (Fig. 11b) as a reliable proof of GCNTs being the species responsible for the light absorption and photocatalytic action in the studied system.

DFT calculations of the intercalated multilayer GCN structure with K, Na, and Cl ions (Li was discarded as contributing negligibly to the change of the interplanar distance) showed that both the intercalated GCNTs and bulk GCN have similar bandgaps and positions of CB (−1.17 V *versus* NHE, pH 7) and VB (+1.53 V *versus* NHE, pH 7). At the same time, the spatial localization of photogenerated electrons and holes was found to differ strongly between the two compounds (Fig. 11b). The electron and hole density distributions in CB and VB of bulk GCN are rather homogeneous, while the electrons were found to be confined to the intercalated area and holes kept far from this area in the intercalation compound, which allows a much higher probability of charge separation and subsequent chemical reactions to be expected for the latter case. An enhanced charge carrier mobility in the intercalated GCNTs is additionally supported by its about 70 times higher electrical conductivity as compared to that of bulk GCN.^94^

The polyheptazine/polytriazine homojunctions produced by tuning the temperature of the melamine conversion in the molten salt environment showed narrower bandgaps as compared to both pure GCN and PTI as a result of strong electronic interactions in such composites and an advanced visible light harvesting capability.^96^ The tubular GCN/PTI homojunctions exhibited also an advanced photocatalytic activity in water splitting as compared to that of bulk GCN as well as both GCNTs and PTI (Fig. 11d) with the highest apparent QY achieved being 2.88% at 420 nm, 0.59% at 450 nm, and 0.16% at 500 nm.^96^

Mesoporous GCNTs produced from urea microrods exhibited a high photocatalytic activity in hydrogen evolution from aqueous TEA solution in the presence of Pt NCs with an apparent QY of 6.3% at 420 nm.^102^

##### (II) GCNTs produced from supramolecular assemblies

**Fig. 12:**
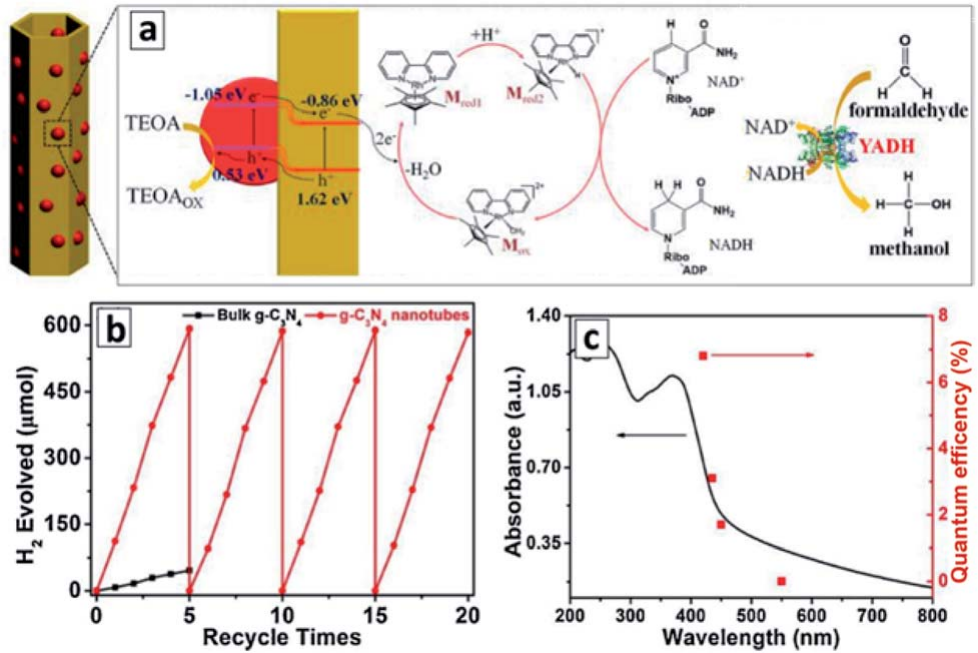
(a) Scheme of the photocatalytic events in the system comprising GCNT/P tubular composite, metal-complex electron mediator, NAD^+^/NADH couple, and formaldehyde as a conversion substrate. Reprinted and adapted with permissions from ref. 103. Copyright (2019) American Chemical Society. (b and c) Kinetic curves (b) and quantum efficiencies (c) of the photocatalytic hydrogen evolution in the presence of GCNTs. Reprinted with permissions from ref. 110. Copyright (2018) Elsevier.

The GCNTs decorated with red phosphorus NCs by the vapor deposition exhibited photocatalytic properties in the generation of reduced nicotinamide cofactor NADH (nicotinamide adenine dinucleotide), which is an essential hydrogen source for many enzyme reduction reactions and is considered as a promising hydrogen carrier for various applications.^103^ The phosphorus NCs with a bandgap of about 1.6 eV performed as a spectral sensitizer for a wider-bandgap GCN (*E*_g_ = 2.7 eV) absorbing the visible light down to 780 nm and injecting photogenerated electrons into GCN tubes as well as accepting holes photogenerated in the GCN tubes upon the photoexcitation with shorter-wavelength light (Fig. 12a). The electrons were transferred to a mediator – pentamethylcyclopentadienyl rhodium bipyridine which then reduced NAD^+^ to NADH. The photocatalytic cycle was completed by the TEA oxidation by the VB holes of the phosphorus NCs.

A combination of this photocatalytic system with an appropriate enzyme – yeast alcohol dehydrogenase (YADH) allows the reduced NADH to be used as a hydrogen source for the reduction of formaldehyde to methanol.^103^ Such system can potentially be coupled to other photocatalytic systems producing CH_2_O from CO_2_ or used independently for the formaldehyde-to-methanol conversion as one of the ways of the solar light energy storage.

The tubular GCN photocatalyst showed a high stability and reproducibility of the photocatalytic properties in cyclic water reduction experiments (Fig. 12b). These features along with the sensitivity to a large portion of visible light to 500–550 nm (Fig. 12c) with an apparent H_2_ QY of 6.8% at 420 nm (ref. 110) show good perspectives of GCNTs for sustainable solar energy conversion technologies.

The superiority of GCNTs produced from supramolecular assemblies in the photocatalytic water splitting under the illumination with visible light over conventional bulk GCN was supported by other reports.^112–114,116,118,119,121,123,128^ An effect of extended nπ* absorption observed in GCNTs synthesized from supramolecular assemblies of urea and oxamide^124^ allowed a broader portion of the visible light to be harvested and an apparent QY of the photocatalytic hydrogen evolution of 1.3% at 525 nm to be achieved, which is by an order of magnitude higher than for that of conventional bulk GCN.

Graphene quantum dots were assumed to act as a spectral sensitizer of tubular GCN produced from supramolecular M–CA assemblies allowing the visible light with *λ* > 600 nm to be harvested, however, no photoaction spectrum was reported supporting this assumption.^116^

Holey GCNTs produced from supramolecular M + CA assemblies revealed a much higher photocatalytic activity in hydrogen evolution from aqueous solutions of lactic acid as compared to that of bulk and nanosheet GCN attributed to the mesoporous character of the tube walls, which facilitates the mass transport and the possibility of a directional charge transport along the main tube axis favoring the spatial separation of the photogenerated charge carriers.^107^ The same factors were assumed to be responsible for a 17 times higher photocatalytic activity of ionic-liquid-templated GCNTs in hydrogen evolution from TEA solutions as compared to that of bulk GCN.^108^

Nitrogen-rich GCNTs produced from supramolecular assemblies of melamine with hydroxylammonium chloride showed excellent adsorption capacity toward carbon dioxide as well as a high photocatalytic activity in the CO_2_ reduction both by water and additional sacrificial donors.^122^ The rate of the photocatalytic CO evolution over such GCNTs was found to be 17 and 15 times higher than over bulk GCN and titania P25.^122^

The introduction of nitrogen vacancies in GCNTs by post-synthesis thermal treatment was found to result in a considerable increase in their photocatalytic activity in the CO_2_ reduction to CO by water vapors without additional electron donors or co-catalysts.^105^ Due to the complex effect of N vacancies on the photophysical properties of GCNTs, both in extending the light sensitivity range and in providing the traps states for the photogenerated electrons, the highest photocatalytic activity is observed for an intermediate population of vacancies, at which the CO_2_ conversion rate achieves almost 44 μmol g^−1^ h^−1^, surpassing by an order of magnitude the photoactivity of bulk GCN with no N vacancies.^105^ This report, however, did not provide a comparison of bulk and tubular GCN with the same conditions of post-synthesis thermal treatment, which hinders a separate evaluation of the effect of N vacancies and the tubular shape on the enhancement of the photocatalytic activity. In contrast, the formation of carbon and nitrogen vacancies in GCNTs induced by the presence of strongly oxidizing nitric and iodic acids, was shown to enhance the photocatalytic water reduction to H_2_ by a factor of 3.64 as compared to that of similar tubes produced with no acids present.^109^

A strong, more than an order of magnitude, enhancement of the photocatalytic water reduction to H_2_ was observed for GCNTs produced from M + CA supramolecular assemblies as compared to bulk g-C_3_N_4_ and assigned both to the tubular structure (high surface area, directed charge flow along the tube axis and light trapping effect) and to the presence of N vacancies acting as charge carrier traps.^110^

##### (III) Scrolled GCNTs

**Fig. 13:**
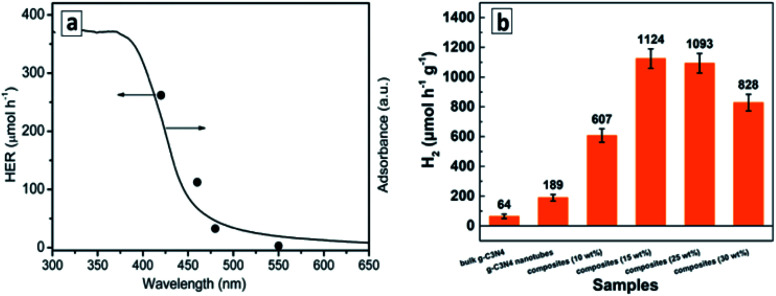
(a) Wavelength-dependent rate of photocatalytic water splitting over GCNTs; (b) rates of photocatalytic hydrogen evolution over GCNT/MoS_2_ composites. Reprinted with permissions from ref. 131 (a) and ref. 138 (b). Copyright (2015) The Royal Society of Chemistry (a) and (2020) Elsevier (b).

The superiority of GCNTs over conventional GCN in photocatalytic water splitting was reported for tube samples produced by anti-solvent-induced scrolling.^131^ The rate of H_2_ evolution from aqueous TEA solutions under monochromatic illumination with different wavelengths is directly proportional to the light absorption of the GCNTs in the corresponding spectral range (Fig. 13a) indicating that a portion of visible light down to 550 nm can be harvested in such a system.^131^ The tubular aggregates of GCN decorated with graphitic carbon dots and Pt NCs showed even more extended spectral range of activity in water splitting, down to 600 nm, and an apparent QY of 10.94% at 420 nm (with a hydrogen evolution rate of 3538 mmol g^−1^ h^−1^) when methanol was used as a sacrificial electron donor.^137^

The GCNTs produced by the scrolling process can be decorated by MoS_2_ nanoflakes, which distribute evenly over the surface of tubes and serve as a co-catalyst for the photocatalytic hydrogen evolution from aqueous TEA solutions.^138^ With no catalysts present, the GCNTs manifest 3 times higher photocatalytic activity as compared to that of bulk GCN. The photoactivity of GCNTs increases strongly upon introduction of MoS_2_ flakes, with a 6-times increase at 15 wt% catalyst (Fig. 13b), but decreases at a higher MoS_2_ content, most probably, due to the light shielding effect.^138^ Individual MoS_2_ flakes showed no activity in this process indicating that it does not contribute to the hydrogen evolution as a photocatalyst and promotes only the secondary processes such as water reduction and recombination of atomic hydrogen.

The Ba-and P-co-doped GCNTs produced by scrolling were reported to be around 14 times more efficient photocatalysts of water splitting as compared to bulk GCN.^140^ DFT calculations showed both dopants to contribute to the bandgap narrowing as well as to a higher delocalization of the GCN LUMO (Lowest Occupied Molecular Orbital) state thus increasing the efficiency of spatial separation of the photogenerated electrons and holes.^140^

The GCNTs produced by shock-freezing induced rolling and modified by bimetallic Ag–Cu NCs^130^ or Pt NCs^136^ revealed a high photocatalytic activity in hydrogen evolution from aqueous solutions of sacrificial donors (triethylamine, TEA, methanol), the efficiency of this process correlating with the oxidation potential of electron donors used.^130^

The photocatalytic water splitting process can be combined with the photocatalytic degradation of potentially dangerous water phenolic pollutants in systems based on GCNTs modified with Pt NCs.^132^ Here, molecular hydrogen can be evolved from aqueous solutions using chlorophenol and nitrophenol as electron donors thus utilizing the water decontamination process for light energy accumulation.

The GCNTs produced by scrolling of the nanosheets, which were formed from melamine/salicylic acid mixtures, showed a pronounced photocatalytic activity in CO_2_ reduction and oxidation of 2-propanol vapors.^144^ The principal product of CO_2_ reduction was methanol forming around 2.5 times faster than in the presence of bulk GCN. The scrolled-up phosphorus-doped mesoporous GCNTs were able to photocatalytically reduce CO_2_ to CO and CH_4_ by a factor of respectively 3 and 14 faster as compared to the bulk GCN.^139^ Additionally, the P-doping of GCNTs favored selective methane formation, the GCNTs showing a CO/CH_4_ molar ratio of 1.3 in contrast to 6.0 for bulk GCN.^139^ This observation indicates a strongly positive role of the mesoporous structure for the electron collection capability of GCNTs and realization of multi-electron processes, which is one of the most recognized challenges in the field of the light-driven CO_2_ conversion to fuels.^16,17,27,31,37,156,157^

Silver- and lanthanum-doped GCNTs produced by a direct ultrasound treatment of protonated GCN nanosheets were found to be efficient photocatalysts of CO_2_ reduction with water vapor (CO_2_ + H_2_O = CO + 3H_2_) and dry reforming of methane (CH_4_ + CO_2_ = 2CO + 2H_2_).^135^ These processes were performed simultaneously resulting in the methane bi-reforming (2CH_4_ + CO_2_ + 2H_2_O = 4CO + 8H_2_), which is a promising route to hydrogen-enriched syngas.

The entire mechanism of this very complex photocatalytic process is still to be explored in detail. The authors^135^ suggested that its key steps are the oxidation of methane and water by highly-reactive photogenerated holes of GCNTs and hydroxyl-radicals, as well as the reduction of water to H_2_ and CO_2_ to CO and CH_3_OH. Methanol can also be produced by the oxidation of CH_4_ by the photogenerated OH˙ radicals, while ethane forms *via* the recombination of 
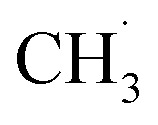
 radicals generated by the methane oxidation with the photogenerated VB holes. The La and Ag dopants are supposed to participate mostly in the steps of CO_2_ reduction with the photogenerated electrons.^135^

The decoration of GCN microtubes with transparent imidazolate ZIF-8 clusters allows the adsorption capacity of GCNTs to be increased toward CO_2_ without compromising their light harvesting capability and thus achieve 3 times more efficient photocatalytic conversion of carbon dioxide into methanol as compared to that of bulk GCN.^159^

The GCNTs produced from M + CA supramolecular assemblies and rich in nitrogen vacancies were found to be excellent photocatalysts of nitrogen reduction at the expense of versatile electron donors (aliphatic alcohols and carboxyl acids) under the illumination with visible light.^126^ Similar to the broadly studied GCN nanosheets,^19,155^ the primary step of the photocatalytic process is the coordination of dinitrogen on the N vacancies, which match perfectly the size of N_2_ molecules resulting in the activation of triple N–N bond and facilitating the nucleophilic attack of CB electrons with the latter also captured by the nitrogen vacancies. The brutto-reaction of the photocatalytic reduction can be expressed as follows: N_2_ + 6e^−^ + 6H^+^ = 2NH_3_. The rate of nitrogen reduction over GCNTs reaches almost 120 mg L^−1^ h^−1^ g^−1^, which is more than 20 times higher than that of bulk GCN.^126^

The GCNTs produced by scrolling and modified with Rh NCs were reported as a visible-light-sensitive photocatalyst of dechlorination of mono-, di-, and tri-substituted chlorophenols into the corresponding alcohols and ketones in aqueous solutions saturated with molecular hydrogen.^134^ In contrast to the more conventional photocatalytic destruction of substituted phenols proceeding through the hydroxylation, subsequent opening of the aromatic rings, and the formation of a mixture of oxycarboxylic acids,^50,51^ the reported process allows the homogeneous dissociation of C–halogen bonds on the surface of Rh NCs to be achieved and coupling of Cl and phenol radicals with hydrogen atoms to form HCl and hydrogenated alcohols and ketones with a selectivity reaching 80–90%.^134^

**Fig. 14:**
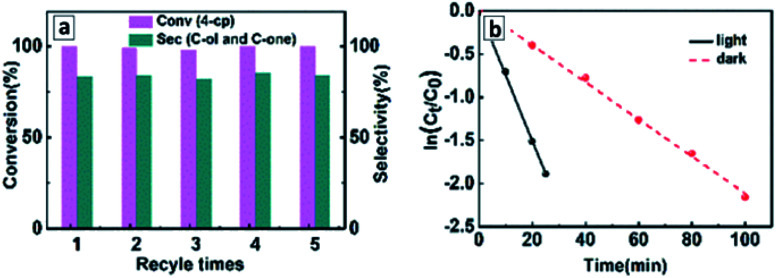
Conversion and selectivity of 4-chlorophenol conversion over Rh-decorated GCNTs in a cyclic experiment (a) and kinetic curves of the process under illumination and in the dark (b). Reprinted with permissions from ref. 134. Copyright (2019) The Royal Society of Chemistry.

The GCNT/Rh composite shows a high stability and recyclability (Fig. 14a) and can be used both as a photocatalyst and as a catalyst with no additional illumination applied (Fig. 14b).^134^ The latter observation demonstrates that Rh NCs play an important role in promoting the dissociation of H_2_ into atomic hydrogen available on the metal NCs for these transformations both under illumination and in the dark. In general, this report shows a promising way for the development of new (photo-)catalytic syntheses of high-added-value chemicals using solar irradiation.

The mechanism of such transformations is still to be explored and includes, most probably, the photocatalytic reduction of water to atomic hydrogen on Rh NCs, rather than direct participation of photogenerated GCN electrons in breaking C–Cl bonds of the phenolic substrates as assumed in ref. 134.

#### Oxidative photocatalytic processes with the participation of GCNTs

The oxidative photocatalytic processes with the participation of GCNTs encompass decomposition of phenolic compounds and organic dyes as well as the gas-phase transformation of NO and CO to deeply oxidized compounds.

Tetragonal GCNTs produced by the molten salt process were tested as a durable and visible-light-sensitive photocatalyst of the degradation of phenol and methylene blue dye.^100^ GCN whiskers synthesized by a similar approach were found to be highly luminescent and applied to selectively detect tetracycline in aqueous solutions by a PL quenching mechanism^99^ and thus the sensing and decomposition of tetracycline can potentially be achieved simultaneously in a single light-sensitive GCNT-based system.

Sodium- and sulfur-co-doped Pluronic-templated GCNTs were probed as a visible-light-sensitive photocatalyst of the decomposition of *p*-chlorophenol and rhodamine B demonstrating high durability and reusability in multiple degradation cycles.^92^ Trapping studies of reactive oxygen containing intermediates showed the superoxide radical O_2_^−^˙ generated by CB electron transfer to oxygen to be the main actor participating in the oxidation of both substrates, while OH˙ radicals produced by VB holes play only a minor role in these processes.^92^ This pathway of the oxidative reaction is of general character for GCNT-mediated photocatalytic oxidative reactions.

GCNTs produced from supramolecular assemblies demonstrated a markedly superior photocatalytic activity in the degradation of organic dyes^111^ and phenols^123^ as compared to bulk GCN. Templated GCN material with entangled tubular structure was reported to be a durable and active photocatalyst of tetracycline and methyl orange degradation.^101^ GCNT filled with ZnO NCs and modified with reduced graphene oxide sheets as an electron mediator showed a high photocatalytic activity in the degradation of a water pollutant – deoxynivalenol.^86^ Chlorophenol and nitrophenol were successfully decomposed with a simultaneous release of hydrogen in a photocatalytic system based on Pt Nc-modified GCNTs.^132^ The tubular GCN/Ag composites produced by HTT-induced scrolling were tested as photocatalysts for the degradation of methyl orange under illumination with visible light.^133^

GCNTs produced from melamine and urea *via* the supramolecular assembling were reported to perform as a very efficient photocatalyst of the oxidation of NO with molecular oxygen.^125^ This particular mode of synthesis generates carbon vacancies, which serve as adsorption and activation centers for both NO and O_2_ molecules as supported by DFT modelling.^125^

Simultaneous scrolling of GCN nanosheets and reduction of Au and Pd salts was applied to form Au–Pd NC-decorated GCNTs showing a promising high catalytic activity in the CO oxidation to CO_2_ by molecular oxygen.^143^ The tubes provide a carrier with a high surface area (around 320 m^2^ g^−1^) for the catalytically active metal NCs as well as ensure the electronic contact among the distant Au–Pd NCs allowing for an efficient transfer of electrons between adsorbed CO and O_2_ molecules.

### 3.3 Photochargeable capacitors based on GCNT membranes

The membranes formed by aligned and closely packed GCNTs were found to reveal a collective behavior resulting in new properties not observed for individual tubes. The phenomenon of photoinduced charging of GCNT membranes due to ionic flows through the membrane channels stimulated by the photoinduced charge carrier separation in the tubes and reported recently by the group of M. Antonietti^82,87,88^ is a vivid example of such collective behavior, which can open new venues of GCNT applications in light-energy storage technologies.

**Fig. 15:**
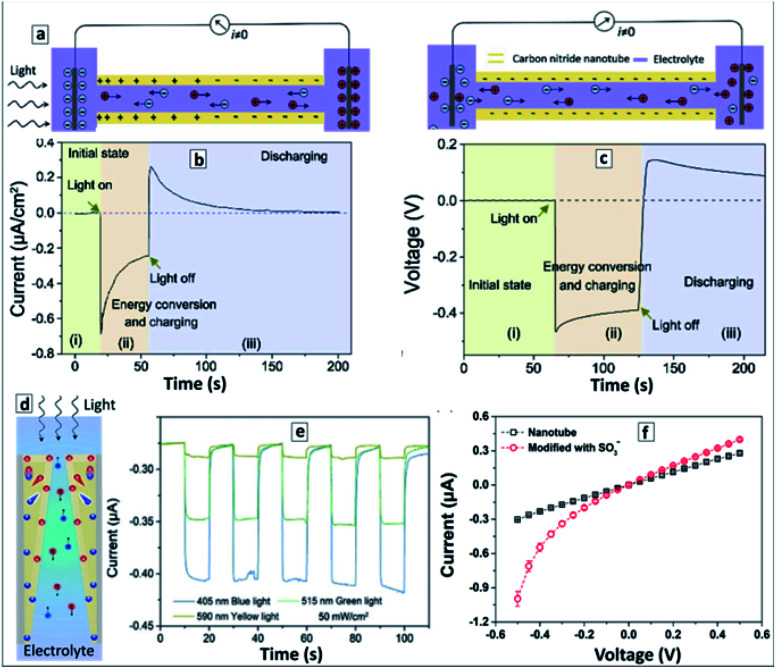
(a) Schematic representation of photoinduced charging (left panel) and dark-stage discharging (right panel) of a photochargeable GCNT-based ionic capacitor. (b and c) Current–time (b) and voltage–time (c) curves on initial, photo-charging, and discharging stages. Reprinted with permissions from ref. 88. Copyright (2020) Elsevier. (d) Schematic representation of photocharging of an asymmetric GCNT membrane. (e) Photocurrent responses of the ionic photodetector based on an asymmetric GCNT membrane under illumination with different light (blue, green, yellow) at −0.5 V. Reprinted and adapted from ref. 82. (f) Current–voltage curves before (black squares) and after (red circles) modification of a GCN membrane with AHPA. Reprinted and adapted from ref. 87.

A light-chargeable ionic capacitor was constructed with a GCNT membrane placed between two collector electrodes (Fig. 15a).^87^ The unilateral (only from one side) illumination of the GCNT membrane filled with an aqueous electrolyte into the absorption band of GCN (*hv* > 2.7 eV) results in the photoinduced spatial separation of the CB electrons and VB holes. The charge separation within the photoexcited GCN tubes is facilitated by the elongated shape of the tubes and different mobilities of electrons and holes, resulting in the migration of electrons to the tube ends opposite to the illumination source. The charge separation, in turn, induces the diffusion of cations and anions to negatively and positively charged ends of the GCNT membrane. In this manner, the cations and anions accumulate on porous carbon electrodes placed at different electrolyte compartments separated by the GCNT membrane, forming a capacitor, which starts to discharge once the illumination is turned off (Fig. 15b and c). The charging/discharging currents and voltages can be scaled up through parallel (current) and series (voltage) connection of several devices.^88^

A similar effect of photoinduced directed ion transport was exploited in a self-powered photodetector based on a GCNT membrane with asymmetric (concave) channels (Fig. 15d).^82^ The unilateral illumination of such membranes from the side with smaller channel diameter results in a non-uniform generation of charge carriers, which are then separated due to differences in mobility and induce ionic diffusion currents in the electrolyte to compensate for the charge distribution inhomogeneity in the GCNT membrane. The resulting photogenerated ionic current is directly proportional to the illumination intensity and increases with a decrease of the excitation wavelength (from “yellow” 590 nm to “green” 515 nm to “blue” 405 nm, Fig. 15e).^82^

Such photodetector demonstrated short light-on response and light-off relaxation times of 0.05 s and 0.85 s, respectively, as well as high values of spectral selectivity, signal-to-noise ratio (around 5000), and sensitivity, combined with a high stability and the self-power capability to operate without an external bias.

The effect of directed ionic transport can also be achieved in dark conditions after a non-uniform modification of GCNT membranes with molecular species that can be charged *via* protonization/deprotonization in an electrolyte. In particular, the photoinduced grafting of 3-allyloxy-2-hydroxy-1-propanesulfonic acid (AHPA) anions or allylamine (AA) inside the GCNT membrane channels results in a gradual modification of the membranes with negatively charged (AHPA anions) or positively charged (protonated AA cations) residuals, their concentration decreasing from the illuminated edge of the membrane into the depth of the tubes.^87^ Such modification imparts GCNT membranes with properties of a diode. The AHPA-modified GCNT membrane allows for selective transport of anions under a negative bias (−0.5 V) resulting in an increased negative current (Fig. 15f), while no differences in the behavior of the modified membrane can be observed under a positive bias (+0.5 V). The AA-modified GCNT membranes show a reverse behavior, selectively conducting cations through the channels under a positive bias and showing no ion rectification behavior under a negative bias.

### 3.4 Light-driven locomotion as a highly promising emerging application of GCNTs

Motion of micro- and nano-objects generated by chemical transformations in the surrounding medium or an external force such as magnetic field, illumination, directed heating, or ultrasound is a topic of highly concentrated interest due to a variety of lucrative applications that can be realized with such micromotors, ranging from automated sensing and collection/decomposition of dissolved pollutants to micro-surgery and highly targeted drug delivery.^160–167^ At that, the utilization of light as the external force is of utmost importance, because light can provide the energy for the locomotion and simultaneously direct the photoactive micromotors along (or opposite to) the light beam. The mechanisms of the light-induced motion can be very versatile, including thermal effects, ionophoresis induced by the photoinduced charge separation as well as a mechanical thrust generated by the formation of bubbles as gaseous products of photoinduced reactions on the surface of a micromotor.^166–169^

GCN has all features necessary for the design of light-driven micromotors, including a versatility of possible anisotropic shapes (rods, wires, tubes, *etc.*) and a pronounced photochemical activity. At the same time, the issue of light-driven motion of GCN-based micromotors remains still unexplored with a single report of the group of M. Pumera^115^ available showing the feasibility of photocatalytic bubble-ejection based motion of GCNTs at the expense of the oxidation of hydrogen peroxide, which is typically applied as a fuel for catalytic micromotors.^160–165^

**Fig. 16:**
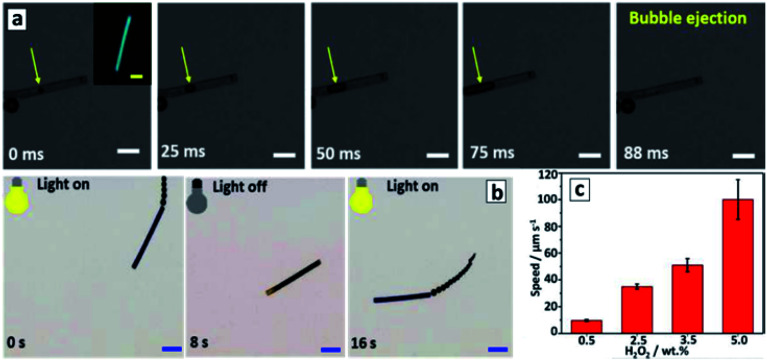
Panel (a): time-lapse images of bubble formation and ejection from a GCNT micromotor under visible light irradiation in the presence of 20 wt% H_2_O_2_ and 0.25 wt% sodium dodecylsulfonate, scale bars: 10 μm. Panel (b): motion of a GCNT micromotor controlled by switching the light on/off/on. (c) Average speed of GCNT micromotors at different concentrations of hydrogen peroxide fuel. Reprinted and adapted with permission from ref. 115. Copyright (2018) American Chemical Society.

Tetragonal shaped GCNTs produced from supramolecular M + CA assemblies demonstrated the capability of directional motion as a result of the photocatalytic decomposition of H_2_O_2_ inside the tubes and ejection of oxygen bubbles.^115^ The average diameter of GCNTs, around 10 μm, is by far larger than the critical minimal tube diameter necessary for the efficient nucleation and ejection of O_2_ bubbles (around 600 nm),^161–163,165^ while its length, about 70 μm, and intense blue photoluminescence allow to track the light-induced motion with optical and fluorescence microscopy (Fig. 16).

As the rate of O_2_ bubble nucleation on the inner side of tubes is higher than on the outer side,^161–163,165^ an oxygen bubble is forming inside the tube (Fig. 16a) and is ejected from one of the tube ends resulting in a propulsion with a rate depending on the light intensity and H_2_O_2_ concentration, reaching 72 μm s^−1^.^115^ As soon as the first bubble is ejected from the tube the symmetry of the bubble formation becomes broken and subsequent bubbles continue to be ejected from the same tube end.^161–163^ These events result in a continuous motion till the light is turned off and resumes after the light is turned on again (Fig. 16b). Hydrogen peroxide reduction by the photogenerated CB electrons of GCNTs is proposed^115^ as the principle mechanism of fuel consumption and O_2_ bubbles generation in this system. At the same time, the participation of VB holes cannot be excluded as well resulting in a cyclic photocatalytic action of GCNTs similar to metal ion photocatalysts of the Fenton process.^50^

The rate of light-induced motion of GCNTs was found to increase up to 100 μm s^−1^ in the presence of Cu^2+^ ions (Fig. 16c), however, no motion was observed in the presence of only Cu^2+^.^115^ The copper-induced motion acceleration can be interpreted as a result of the photocatalytic Cu^2+^ reduction to Cu^0^ by photogenerated CB electrons, the Cu^0^ deposits acting as a co-catalyst of H_2_O_2_ decomposition. Alternatively, the adsorbed Cu^2+^ can aid in decomposing H_2_O_2_ through a Fenton-like process.^50^ As the Cu^2+^ adsorption results in a strong PL quenching of GCNTs, the authors suggested that GCNT micromotors can be used as self-reporting sensors of copper pollution in water with a built-in capability of simultaneous recovery of the pollutant.^115^

### 3.5 Electrochemical and electrocatalytic activity of GCNTs and related composites

The number of reports on the electrochemical activity of GCNTs is rather scarce and focused on sensing systems with GCNTs as a conductive carrier. This is in a marked contrast to the progress with GCN nanosheets^13,15,38,41–44^ and shows that there still is a great potential in GCNT electrochemistry to be discovered.

GCNTs synthesized by hydrothermal scrolling of nanosheets were widely used as a platform for the formation of highly-sensitive and selective molecularly-imprinted electrochemical sensors.^170–172^ The imprinting was performed by multiple voltamperic cycles of a GCNT-containing electrode in a solution of phenol and a target molecule (melamine, atrazine, bilirubin, *etc.*). Phenol was gradually oxidized during the cyclic voltammetry forming a polymerized film on the electrode surface. Due to the formation of van der Waals and/or electrostatic bonds between the analyte and phenol, the analyte was captured on the surface during the electropolymerization of phenol and then washed out by the NaCl electrolyte, thus leaving a characteristic adsorption site, which imparted the electrode with a selectivity toward the given analyte. This approach was applied to produce electrosensors for melamine (detection limit 1.0 × 10^−11^ M),^172^ atrazine (detection limit 1.5 × 10^−13^ M),^170^ bilirubin (detection limit 3.0 × 10^−13^ M),^171^ and ractopamine (detection limit 1.0 × 10^−13^ M).^173^

GCN tubes also revealed electrocatalytic activity in the electrooxidation of methanol.^89^

## 4 Formation and properties of polytriazine based GCNTs

**Fig. 17:**
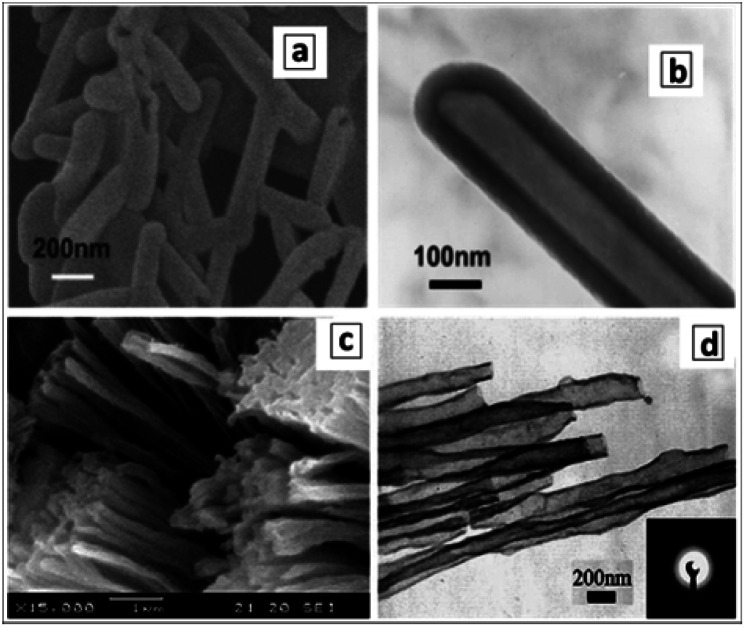
SEM (a and c) and TEM (b and d) images of triazine NTs. Reprinted with permissions from ref. 174 (a and b) and ref. 175. Copyright (2004) The Royal Society of Chemistry (a and b) and (2006) Elsevier.

Polytriazine-based tubes are studied to a much lesser extent as compared to their polyheptazine allotrope, their syntheses mostly confined to reductive conversions of triazine precursors in rather harsh conditions. In particular, closed-end triazine NTs were produced by a hydrothermal treatment of 1,3,5-trichlorotriazine and sodium azide in benzene at 220 °C.^174^ The tubes revealed highly disordered walls with a thickness of 20–50 nm and an inner tube diameter in the range of 50–100 nm (Fig. 17a and b).^174^

Bundles of open-end triazine NTs were produced by reducing cyanuric chloride (C_3_N_3_Cl_3_) with sodium at 230 °C and 1.8 MPa in an autoclave with NiCl_2_ as a catalyst precursor.^175,176^ The tubes are a few to tens of micrometers long with a diameter ranging between 50 and 100 nm (Fig. 17c and d). A tentative mechanism of the tube growth includes reduction of NiCl_2_ to metallic Ni islands on the surface of sodium granules followed by the base-growth of triazine NTs on Ni islands *via* the reduction of cyanuric chloride to C_3_N_3_ clusters with sodium.^175,176^

Similar to the case of base-growth of carbon NTs,^63^ the triazine tube growth proceeds constantly on the surface of the Ni catalyst island resulting in the uniform tubular morphology of the products. Apparently, the diameter of NTs is determined by the size of the Ni catalyst islands.^175^ A study of triazine NTs using electron energy loss spectroscopy (EELS) revealed the presence of sharply defined π* and σ* fine structure features indicating sp^2^-hybridization of both types (C and N) of constituent atoms. The N/C ratio determined from EELS spectra was found to be 1 consistent with the triazine C_3_N_3_ stoichiometry.^176^

Triazine NT formation was observed at a heat treatment of polytriazine sheets decorated with iron NCs.^177^ The precursor sheets were produced by polymerizing 2,4,6-tricyano-1,3,5-triazine accelerated by CF_3_SO_3_H followed by cyano-group elimination at 400 °C in the presence of ZnCl_2_.^177^ The Fe NC-decorated triazine NTs showed a high electrocatalytic activity in oxygen reduction comparable to that of Pt/C and IrO_2_ electrocatalysts.^177^

## 5 Conclusions and outlook

The present review of the reports on the formation and properties of GCNTs allows their most prominent characteristic features to be highlighted as well as some predictions to be made about the most promising venues of further research in this field.

The formation of GCNTs is typically achieved by three general approaches, including template synthesis to form both individual GCNTs and closely packed GCNT arrays, thermal evolution of supramolecular macro-/microrod-shaped precursor assemblies, and stimulated scrolling of GCN nano-/micro-sheets into tubular entities. Spontaneous formation of GCNTs is also observed and assumed to occur due to flows of gases released from the material subjected to thermal polycondensation. However, this phenomenon has an occasional character and does not allow the morphology of the tubular products to be controlled. In this view, further advances in the synthesis of GCNTs are expected to arise from new template materials, new (and most probably more complex) compositions and geometries of supramolecular assemblies-precursors, and more reliably controlled and versatile approaches to the rolling up GCN nano- and microsheets as well as atomically-thin single polyheptazine layers into tubes and scrolls.

Each of the three major approaches to the synthesis of GCNTs has inherent benefits and special features making them unique but, at the same time, highly complementary to each other in the design of new GCN-based materials. Template approach is perfectly suited for the preparation of *GCNT arrays/membranes* allowing both the morphology and composition of final GCNT membranes to be precisely controlled. At the same time, an additional step of template removal is obligatory in most of the reported template-assisted protocols. The synthesis through supramolecular assemblies is perfectly suitable for the preparation of individual GCNTs with a tailored shape. The morphology of rod-like supramolecular assemblies which convert to GCNTs upon polycondensation is governed mostly by the chemical structure of molecular precursors opening almost unlimited opportunities for a combinatorial design of the assembly composition and, thus, of the shape and wall composition of final GCNTs. Rolling-up approach provides unique opportunities for the modification of GCN sheets with molecular and nanocrystalline species (dyes, semiconductor and metal nanocrystals, ferments, *etc.*) *prior to the scrolling of sheets into GCNTs* which is especially favourable for the formation of complex functional materials with a highly controlled spatial organization.

The application domain of GCNTs is dominated by their activity as photocatalysts, mostly of light-accumulating endothermic processes, such as water splitting and carbon dioxide conversion, both favored by the relatively high CB potentials of the photogenerated electrons and the versatility of available efficient co-catalysts of multi-electron reductive reactions.

The GCN-based nano- and microtubes show generally a much higher photocatalytic activity in a number of processes as compared to bulk GCN samples produced in the same calcination/doping conditions. These processes include water reduction to H_2_ at the expense of the oxidation of various electron donors, reduction of CO_2_ with water as well as decomposition of phenolic compounds and dyes. Typically, the GCNTs are characterized by slightly narrower bandgaps and less negative CB levels as compared to bulk GCN. In this view, the advanced photocatalytic activity is assumed to originate from the high surface area of the GCNTs and the anisotropy of their shape, which makes it challenging to evaluate the contribution of the shape factor separately, because GCNTs and bulk GCN typically differ strongly in their specific surface area. In the systems aimed at carbon dioxide reduction, the advanced CO_2_ adsorption capacity of GCNTs per surface unit as compared to the conventional bulk of nanosheet GCN additionally contributes to the higher photoactivity. Hence, the effects of a directed flow of the photogenerated charge carriers along the main axis of GCNTs, the possibility of the charge carrier migration along the network of nanocrystals or nanosheets forming the tube walls in the case of porous GCNTs as well as the effect of light trapping arising from multiple light reflection and scattering inside the tubes can (and most probably do) all contribute to the enhanced photocatalytic activity of GCNTs, but still are to be evaluated critically and consistently.

At the same time, there are several factors that can enhance the photocatalytic properties of GCNTs, which can not be realized in the case of bulk and mesoporous GCN or GCN nanosheets. First, the reactants coming into the inner void of the nanometer- and especially micrometer-long GCNTs become trapped and forced to multiple interactions with the inner surface of the tubes, while no such limitations can be found in their interactions of discrete GCN crystals or nanosheets. This trapping effect can strongly increase the probability of multi-electron processes, which are crucial, for example, in the reduction of CO_2_ to valuable products (4e^−^ for HCHO, 6e^−^ for CH_3_OH, 8e^−^ for CH_4_), in the 6e^−^ N_2_ reduction to ammonia, or in selective multi-electron (2e^−^ or 4e^−^) reduction of organic compounds. In this view, a proper modification of the inner surface of GCNTs with co-catalysts and electron mediators can produce unique photocatalytic blocks with multi-electron reducing capability. The external surface of GCNTs remains still available and can be additionally modified with spectral sensitizers and co-catalysts of parallel oxidative reactions of VB holes, thus, expanding the range of harvested visible light and suppressing the electron–hole recombination, respectively.

A second unique factor (which is still hypothetic but rather feasible) that can be realized in the case of GCNTs, is the total spatial separation of reductive and oxidative branches of the photocatalytic process. As a classical semiconductor photocatalyst, photoexcited GCN always induces simultaneously the reduction of acceptor substrates by CB electrons and the oxidation of donor substrates by VB holes. The total efficiency of the photocatalytic process depends on a balance between both processes, because of the possibility of recombination between radical intermediates (cation-radicals and anion-radicals), reverse reactions between final products as well as charging bottlenecks when one of the charge carrier types is consumed much slower than the other one. The main challenge is to design the photocatalyst in such a way that the co-catalysts of the reductive and oxidative branches get separated and deposited on different surfaces (inner and outer) of the GCNTs. This aim can be potentially achieved by carefully introducing co-catalysts (or their precursors) in different stages of the GCNT synthesis or by selective etching procedures. With the co-catalysts of reductive and oxidative semi-reactions separated, each GCNT will act as a micromembrane, generating a directed flow of electrons from donating to accepting substrates and totally suppressing possible interactions between intermediates.

Finally, the third unique factor determining the specific photocatalytic behavior and applications of GCNTs is the spatial selectivity of nucleation of gaseous products of photoprocesses due to differences in the energy of the outer and inner surfaces of the tubes. This feature promotes the preferential formation of gas bubbles on the inside rim of the tube ends and the bubble ejection results in the motion of the entire tube in the opposite direction.^166–169^ A pioneering report of M. Pumera *et al.*^115^ on the light-induced locomotion of water-suspended GCNTs at the expense of the oxidation of hydrogen peroxide fuel showed perspectives of this material combining relatively high motion speeds, sensitivity to the visible light, easily functionalizable surface, and the possibility of tracking the moving GCNTs by using their inherent strong photoluminescence. Further work in this direction can be versatile and expected to bring new ideas and materials to the field of locomotion of microobjects. This work includes a search for new co-catalysts of the decomposition of fuel and acceleration of the motion, search for new fuels with a higher affinity to GCNTs, design of spectrally sensitized GCNTs responsive to visible and near-infrared ranges of illumination as well as GCNT-based microbots capable of controlled picking, moving, and releasing of micro-cargos.

The feasibility of light-induced locomotion^115^ was shown for micrometer-wide GCNTs produced by the supramolecular assembly approach. This synthetic approach has a great potential, but remains still far from the maturity stage, provides only a limited means of control over the morphology (length, diameter, wall thickness, and porosity) of the GCNTs, and allows only a post-synthesis modification of the tubes with additional potential co-catalysts, sensitizers, cargo holders, *etc.* In this view, we would put much higher expectations into *the scrolling of pre-synthesized GCN nano- and microsheets into tubes as a much more versatile and potent route to light-driven micro-motors*.

The array of reported data on GCN nano- and microsheets is uncomparably larger than the pool of reports on GCNTs, providing a lot of various possibilities of synthesis and modification of the GCN sheets prior to scrolling into the final GCNTs. The morphology (lateral size, thickness, porosity) of GCN sheets as well as their chemical structure (the presence and density of dopants and vacancies) can be controlled both by the polycondensation conditions and by the mode of post-synthesis treatment. The decoration of the micro-motors with appropriate co-catalysts, surface modifications, electron mediators, and spectral sensitizers can be performed both before the scrolling into the tubes and after the scrolling procedure, providing unique flexibility and versatility of the micro-motor design.

Along with being a classical semiconductor photocatalyst, GCN still remains an organic compound with the entire functionality of modern organic chemistry available for the modification of the polyheptazine layers both in the stage of their formation and in post-synthesis treatments. A vivid example of such approach is the introduction of additional carbo- and heterocyclic compounds, which are built into the polyheptazine network during the polycondensation and enhance the aromatic character of the layers expanding their spectral sensitivity to longer wavelengths.^106^ This particular approach was successfully applied by M. Antonietti *et al.* for the synthesis of GCNTs by additions of triazole heterocycles^121^ and additionally stimulated a search for totally new heterocyclic precursors for the formation of polyheptazine and polytriazine layers.^178^

As a layered graphite-like compound, GCN can also be modified by using approaches developed in the chemistry of 2D materials.^158^ This possibility was vividly exemplified by the modification of the electronic properties of GCN by intercalation of sodium and potassium chlorides during the formation of GCN in molten salt eutectics^94,98^ as well as by affecting the morphology and adsorption properties of the intercalated GCN by the ion-exchange-based removal of the intercalates.^95,97^

Membranes formed by close-packed GCN nano- and micro-tubes appear to be an excellent material for light-energy harvesting and storage, electrochemical and other applications, which can compete in the domains typically occupied by anodized titania membranes.^179–187^ Similar to mesoporous anodized titania membranes, GCNT membranes feature easy scalability of the synthesis, mechanical and chemical stability, broad tunability of the morphology (diameter and shape of the pores, distance between the pores, thickness of the membrane, *etc.*), and potential variability of the chemistry of pore walls with excellent charge transport properties, photochemical activity, and sensitivity to visible light.

One of the most interesting features of the GCNT membranes is the possibility of photoinduced charge separation resulting in ionic flows and charging of electrodes places on both sides of the membrane.^88^ This light-induced capacitor charging becomes even stronger if the membrane is composed of asymmetric concave GCNTs^82^ and potentially can be further increased by the membrane modification by charged functional groups.^87^ Taking into account the sustainability and chemical stability of GCNTs, their sensitivity to visible light as well as a high mechanical durability of GCNT membranes, and the feasibility of the synthesis of large enough membranes to be used in commercial devices, this technology of light storage can be of considerable interest and become a competitor to other types of solar charging batteries and capacitors.^49,188^

We should note that such processes of spatial separation of photogenerated charge carriers favored by the elongated shape of the GCNTs and differences in the mobilities of CB electrons and VB holes would inevitably result in *the spatial separation of partial photocatalytic reactions* taking place on both sides of the GCNT membrane. As the VB holes have a lower mobility than CB electrons, the photoinduced oxidation of adsorbed donor substrates by the VB holes is expected to take place selectively on the illuminated side of the GCNT membrane, while the photoinduced reduction of adsorbed electron acceptors will occur predominantly on the other side of the membrane. This property of GCNT membranes, which was vividly exemplified by the above-discussed photocapacitor effect,^82,87,88^ allows us to expect that GCNT membranes will manifest much higher photocatalytic activity as compared to the individual GCNTs and a far superior photoactivity in comparison with that of bulk GCN.

An important consequence of such spatial separation of the oxidation and reduction half-reactions of a photocatalytic process could be an enrichment of the membrane sides with the products of these processes, such as, for example, H^+^ or OH^−^ ions forming during water splitting processes, creating ion concentration gradients and resulting in additional flows through the membrane channels. We envisage that in this way the GCNT membranes could be used as *light-driven ionophoretic pumps* put in motion by flows of solvated ions generated by photocatalytic processes on the different sides of the membrane.

GCNT membranes are expected to serve as excellent scaffolds for other light-sensitive components of solar cells, in particular for organic donors or acceptors, or hybrid perovskite materials, which can be introduced into the inner voids of the membrane and benefit from the directed charge transport along the GCNTs.

The photoinduced modification of the GCNTs with organic functional groups using the inherent photocatalytic activity of GCNTs^87^ is an excellent way of imparting GCNTs with selectivity in photocatalytic reactions and designing membranes for selective photocatalytic organic syntheses^71,189^ or treatment of water polluted with specific substances.

All these potential applications require an arsenal of sustainable and reliable methods of membrane synthesis allowing a precise control over the population and morphology of inner channels, the membrane thickness, as well as the chemical nature of the wall surface.

Template synthesis of GCNT membranes appeared to be the most fruitful synthetic approach, however, the present review shows that the range of templates used for the formation of GCNT membranes is confined most exclusively to AAO membranes with pore channel sizes of tens to a-few-hundreds of nanometers. As the processes of polycondensation of GCN precursors can be adapted to almost any environment we expect that the range of possible “hard” templates can be expanded to other highly ordered materials, which remained up to now behind the focus of interest in the chemistry of GCNs.

One of such materials is zinc oxide which is well reported to form a variety of highly ordered 3D morphologies, including arrays of nano- and microrods, nanowires, and microwhiskers.^190,191^ The morphology of such 3D ZnO microstructures can be flexibly tuned by templated growth, etching techniques, and precise control over the deposition conditions.^190,191^ Zinc oxide is widely reported to form heterostructures with GCN evidencing a good affinity of such template to growing GCN sheets and can be easily removed by mild acidic or basic etching when the formation of a GCNT membrane is finished. Of special interest can be vertical arrays of ZnO nanowires (ESI, Fig. S1a†)^192–197^ or highly-ordered and regularly-shaped nanorods (ESI, Fig. S1b†)^198,199^ which can be used as a template to produce GCN membranes with uniform and evenly distributed pores. Versatile possibilities of templating the GCNT membranes can be provided by highly-ordered silicon-derived microstructures, including arrays of macropores^200–205^ or microwires/microrods.^201,204,206–210^ Similar to the AAO and titania membranes, the morphology of these ordered structures produced by (photo-) electrochemical etching of silicon can be precisely tuned by the etching conditions, electrolyte composition, inherent properties of the silicon substrates, and morphology of a seeding template.

Silicon microrod arrays are typically characterized by a highly controlled microrod shape, diameter, and length as well as distance between the neighboring microrods (ESI, Fig. S1c†)^206,208^ making them an ideal platform for the formation of ordered GCNT membranes. The macroporous silicon membranes combine an extremely precise and regular morphology with a high mechanical robustness allowing microcutting at a preset angle to be performed (ESI, Fig. S1d and e†)^200^ and thus providing a unique and yet unexplored templating possibility to produce GCNT membranes with a gradual thickness. Macroporous Si membranes can maintain a high pore uniformity in unprecedently thick, more than 500 μm,^205^ layers, which is far beyond the limits of the presently used AAO templates.

Of special interest can be *polymeric templates*, which can direct the polycondensation process to the formation of ordered GCNT microchannel arrays and then be naturally eliminated during the calcination without additional etching steps. We envisage that such templates can be produced by stereolithographic approaches yielding highly ordered 3D arrays of polymer microrods or microwires with a spatial resolution of around 1 μm.^211,212^ In this approach, the shape and length of the polymeric wires can be tuned at will potentially allowing arrays of straight (ESI, Fig. S2a†) or curled (ESI, Fig. S2b†) channels to be produced with this method in future GCNT membranes.

The explosive development of various 3D printing technologies provides additional possibilities for the production of highly ordered polymeric templates. Along with the adapted stereolithographic approaches,^213–215^ the 3D printing can be realized using much more affordable technologies such as direct ink writing, electrospinning, electrohydrodynamic printing, selective laser sintering, and lamination^213,214,216,217^ providing spatial *XY* resolution of 10–20 μm depending on the method and ink components.^213–215^

Both individual polymeric tubes and tube arrays can be printed, for example, by inkjet printing or electrospinning (ESI, Fig. S2c†) with a high spatial control over the tube shape and wall thickness. Along with polymers, a vast number of other materials can be used as inks, including various metals and metal salts, hydrogels, silicon, elastomers, *etc.*^213^ For example, an ordered injet-printed array of Ag/AgCl concave microrods for electrochemical applications^218^ with a tip diameter of around 100 μm expanding down to 600 μm at the rod bottom was produced by this printing technique (ESI, Fig. S2d†). This approach can be envisaged as a route to concave GCNT arrays when a polymeric template or other etchable materials will be used to form the template. Alternatively, the GCNT membranes can be directly printed by using 3D printing technologies adapted for the production of inorganic ordered functional materials^216,219,220^ with appropriately designed GCN-containing inks.^220^

Summarizing, the field of GCNTs is well worth the investment of further research efforts and is expected to be as proficient in new materials and applications as the related areas of carbon nanotubes or mesoporous titania nanotubes and nanotube arrays proved to be. We hope that our review and the concluding outlook will promote the interest in GCNTs as an emerging material with brilliant perspectives and contribute to further progress in this promising field.

## Conflicts of interest

There are no conflicts to declare.

## Supplementary Material

RA-010-D0RA05580H-s001
