# Supplementary material for: Graphitic carbon nitride nanotubes: a new material for emerging applications
Source: RSC Adv. 2020 Sep 15;10(56):34059–87. doi: 10.1039/d0ra05580h (PMC9056768; doi:10.1039/d0ra05580h)
Supplement: RA-010-D0RA05580H-s001 [file RA-010-D0RA05580H-s001.pdf]

Electronic Supplementary Material (ESI) for RSC Advances.
This journal is © The Royal Society of Chemistry 2020

Supporting Information

Graphitic Carbon Nitride Nanotubes: A New Material for Emerging Applications

Oleksandr Stroyuk[1,2*], Oleksandra Raievska[2,3,4], Dietrich R.T. Zahn[3,4]

[1]*Forschungszentrum Jülich GmbH, Helmholtz-Institut Erlangen Nürnberg für Erneuerbare Energien (HI ERN), Immerwahrstr. 2, 91058 Erlangen, Germany*

[2]*L.V. Pysarzhevsky Institute of Physical Chemistry, Nat. Acad. of Science of Ukraine, 03028 Kyiv, Ukraine*

[3]*Semiconductor Physics, Chemnitz University of Technology, D-09107 Chemnitz, Germany*

[4]*Center for Materials, Architectures, and Integration of Nanomembranes (MAIN), Chemnitz University of Technology, D-09107 Chemnitz, Germany*

Authors for correspondence:

[*]Dr. Oleksandr Stroyuk, Forschungszentrum Jülich GmbH, Helmholtz-Institut Erlangen Nürnberg für Erneuerbare Energien (HI ERN), Immerwahrstr. 2, 91058 Erlangen, Germany; *e-mail*: o.stroyuk@fz-juelich.de, alstroyuk@ukr.net.

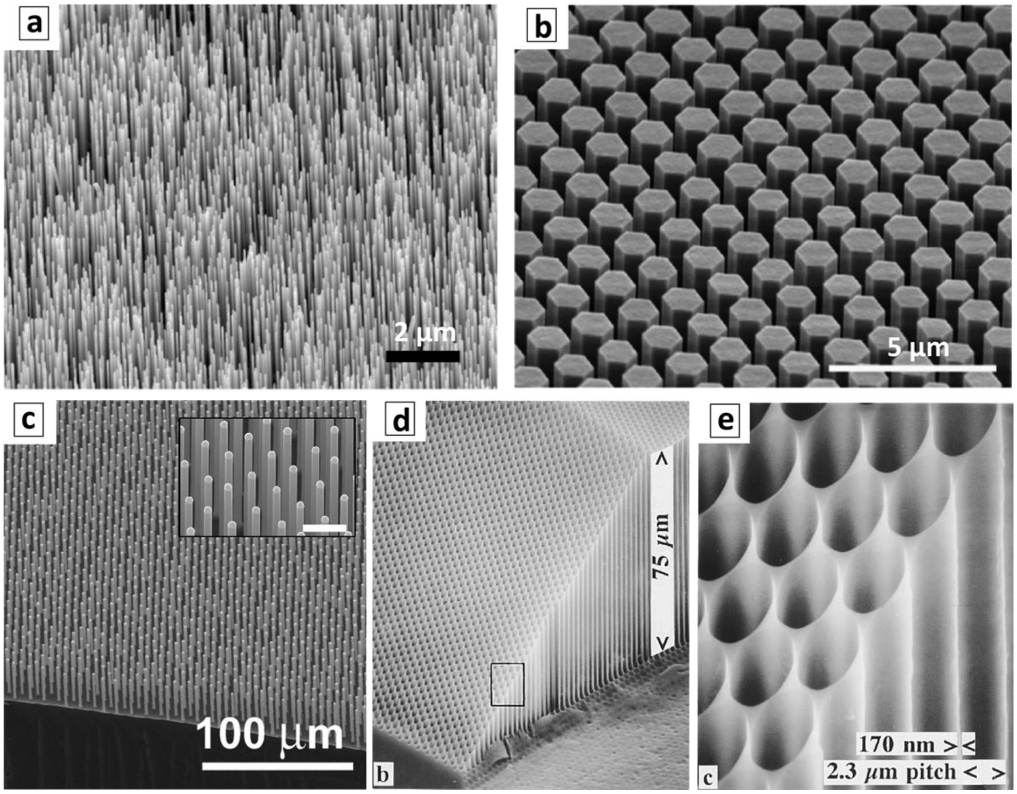

Figure S1. Arrays of ZnO nanowires (a), ZnO nanorods (b), Si nanorods (c), and micropores in Si (d,e). Reprinted with permissions from Ref. 194 (a), Ref. 199 (b), Ref. 206 (c), and Ref. 200 (d,e). Copyright (2007) American Chemical Society (a), (2013) The Royal Society of Chemistry (b), (1996, 2007) American Institute of Physics (c-e).

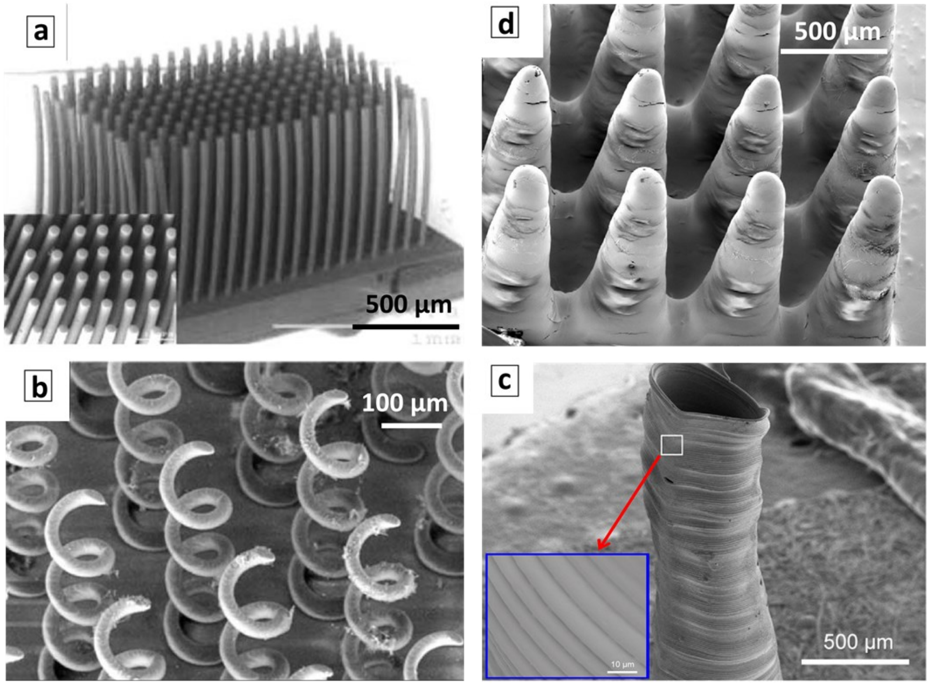

Figure S2. (a, b) Arrays of straight (a) and curled (b) polymeric microrods produced by stereopithoraphy. (c) 3D printed polyvinylidene fluoride microtube (inset shows the wall tube structure); (d) 3D printed arrays of Ag/AgCl microelectrodes. Reprinted and adapted from Ref. 212 (a,b), Ref. 217 (c), and Ref. 219 (d). Copyright (2005, 2012) Elsevier (a,b,d) and (2015) American Chemical Society (c).
